# Production of α-Galactosylceramide by a Prominent Member of the Human Gut Microbiota

**DOI:** 10.1371/journal.pbio.1001610

**Published:** 2013-07-16

**Authors:** Laura C. Wieland Brown, Cristina Penaranda, Purna C. Kashyap, Brianna B. Williams, Jon Clardy, Mitchell Kronenberg, Justin L. Sonnenburg, Laurie E. Comstock, Jeffrey A. Bluestone, Michael A. Fischbach

**Affiliations:** 1 Department of Bioengineering and Therapeutic Sciences and the California Institute for Quantitative Biosciences, University of California, San Francisco, California, United States of America; 2 Department of Biological Chemistry and Molecular Pharmacology, Harvard Medical School, Boston, Massachusetts, United States of America; 3 Diabetes Center and the Department of Medicine, University of California, San Francisco, California, United States of America; 4 Department of Microbiology and Immunology, Stanford University School of Medicine, Stanford, California, United States of America; 5 La Jolla Institute for Allergy and Immunology, La Jolla, California, United States of America; 6 Division of Infectious Diseases, Department of Medicine, Brigham and Women's Hospital, Harvard Medical School, Boston, Massachusetts, United States of America; National Jewish Medical and Research Center/Howard Hughes Medical Institute, United States of America

## Abstract

While the human gut microbiota are suspected to produce diffusible small molecules that modulate host signaling pathways, few of these molecules have been identified. Species of *Bacteroides* and their relatives, which often comprise >50% of the gut community, are unusual among bacteria in that their membrane is rich in sphingolipids, a class of signaling molecules that play a key role in inducing apoptosis and modulating the host immune response. Although known for more than three decades, the full repertoire of *Bacteroides* sphingolipids has not been defined. Here, we use a combination of genetics and chemistry to identify the sphingolipids produced by *Bacteroides fragilis* NCTC 9343. We constructed a deletion mutant of BF2461, a putative serine palmitoyltransferase whose yeast homolog catalyzes the committed step in sphingolipid biosynthesis. We show that the Δ2461 mutant is sphingolipid deficient, enabling us to purify and solve the structures of three alkaline-stable lipids present in the wild-type strain but absent from the mutant. The first compound was the known sphingolipid ceramide phosphorylethanolamine, and the second was its corresponding dihydroceramide base. Unexpectedly, the third compound was the glycosphingolipid α-galactosylceramide (α-GalCer_Bf_), which is structurally related to a sponge-derived sphingolipid (α-GalCer, KRN7000) that is the prototypical agonist of CD1d-restricted natural killer T (iNKT) cells. We demonstrate that α-GalCer_Bf_ has similar immunological properties to KRN7000: it binds to CD1d and activates both mouse and human iNKT cells both *in vitro* and *in vivo*. Thus, our study reveals BF2461 as the first known member of the *Bacteroides* sphingolipid pathway, and it indicates that the committed steps of the *Bacteroides* and eukaryotic sphingolipid pathways are identical. Moreover, our data suggest that some *Bacteroides* sphingolipids might influence host immune homeostasis.

## Abstract 2

A common human gut bacterium, *Bacteroides fragilis*, produces a sphingolipid ligand for the conserved host receptor CD1d and can modulate natural killer T cell activity.

## Author Summary

While human gut bacteria are thought to produce diffusible molecules that influence host biology, few of these molecules have been identified. Species of *Bacteroides*, a Gram-negative bacterial genus whose members often comprise >50% of the gut community, are unusual in that they produce sphingolipids, signaling molecules that play a key role in modulating the host immune response. Sphingolipid production is ubiquitous among eukaryotes but present in only a few bacterial genera. We set out to construct a *Bacteroides* strain that is incapable of producing sphingolipids, knocking out a gene predicted to encode the first enzymatic step in the *Bacteroides* sphingolipid biosynthetic pathway. The resulting mutant is indeed deficient in sphingolipid production, and we purified and solved the structures of three sphingolipids that are present in the wild-type strain but absent in the mutant. To our surprise, one of these molecules is a close chemical relative of a sponge sphingolipid that is the prototypical ligand for a host receptor that controls the activity of natural killer T cells. Like the sponge sphingolipid, the *Bacteroides* sphingolipid can modulate natural killer T cell activity, suggesting a novel mechanism by which *Bacteroides* in the gut might influence the host immune response.

## Introduction

**Figure 1:**
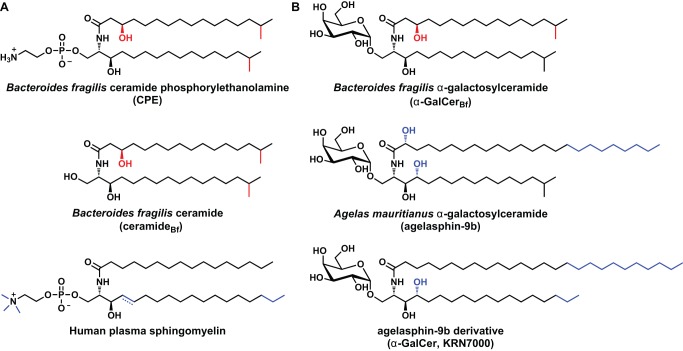
Chemical structures of the *B.* *fragilis* sphingolipids and related molecules. (A) *B. fragilis* produces the phosphosphingolipid ceramide phosphoryl-ethanolamine (CPE, top) and the corresponding free ceramide (ceramide_Bf_, middle), which are similar in structure to the most abundant (4,5-dehydro) and third-most abundant (4,5-dihydro) forms of sphingomyelin in human plasma (bottom). (B) *B. fragilis* produces the glycosphingolipid α-galactosylceramide (α-GalCer_Bf_, top), which is similar in structure to the sponge-derived α-galactosylceramide agelasphin-9b (middle) and a widely used derivative of agelasphin-9b, KRN7000 (bottom). Chemical groups that vary among the molecules in each column are colored red and blue for *B. fragilis* and non–*B. fragilis* sphingolipids, respectively. CPE, ceramide_Bf_, and α-GalCer_Bf_ were each purified as inseparable mixtures of varying lipid chain length. The proposed structures of the most abundant species are shown here.

Sphingolipids and their breakdown products modulate a variety of eukaryotic signaling pathways involved in proliferation, apoptosis, differentiation, and migration (Figure 1). Although sphingolipids are ubiquitous among eukaryotes, few bacteria produce them [1]. The genus *Bacteroides* and its relatives are an important exception; 40%–70% of the membrane phospholipids of these prominent symbionts are sphingolipids [2],[3]. While the structures of several *Bacteroides* sphingolipids have been solved, the full repertoire of these molecules has not yet been defined [1]–[19]. Here, by systematically exploring the sphingolipid repertoire of *Bacteroides fragilis*, we show that this gut commensal unexpectedly produces an isoform of α-galactosylceramide, a sponge-derived sphingolipid that is the prototypic ligand for the host immune receptor CD1d.

## Results and Discussion

### Bioinformatic Insights into *Bacteroides fragilis* Sphingolipid Biosynthesis

To gain insight into the potential role of *Bacteroides* sphingolipids in mediating microbiota–host interactions, we set out to define the complete set of sphingolipids produced by *Bacteroides fragilis* NCTC 9343 [20], a genome-sequenced, genetically manipulable human gut isolate. Reasoning that a chromatographic comparison of lipid extracts from wild-type *B. fragilis* and a sphingolipid-deficient mutant would reveal the complete set of *B. fragilis* sphingolipids, we began by attempting to identify genes involved in *B. fragilis* sphingolipid biosynthesis. We took a candidate gene approach, hypothesizing that the *Bacteroides* sphingolipid pathway would harbor homologs of the eukaryotic pathway [17]. BLAST searches of the *B. fragilis* genome using the *Saccharomyces cerevisiae* sphingolipid biosynthetic enzymes as queries yielded two hits encoded by adjacent genes: BF2461, a putative serine palmitoyltransferase, and BF2462, a putative sphinganine kinase.

Bioinformatic analysis suggested that BF2461, like its yeast homolog, is a pyridoxal-phosphate-dependent α-oxoamine synthase that conjugates serine and a long-chain acyl-CoA to form 3-dehydrosphinganine. In eukaryotes, this serves as the first committed step in the sphingolipid biosynthetic pathway. We therefore predicted that a Δ2461 mutant would be completely deficient in the production of sphingolipids. The eukaryotic homolog of BF2462, sphingosine kinase, phosphorylates sphingosine to form sphingosine-1-phosphate (S1P). Given that this reaction diverts the flux of the sphingosine base away from ceramide and toward S1P, we hypothesized that a Δ2462 mutant would produce a higher titer of mature sphingolipids than the wild-type strain.

### Using Genetics and Chemistry to Define the *B. fragilis* Sphingolipid Repertoire

We constructed a mutant harboring a deletion of BF2461 (Δ2461) (see S1.8 in Supporting Information S1). Although we obtained co-integrates for the BF2462 mutant, double crossover mutants were never obtained despite repeated attempts to screen through thousands of colonies, suggesting that BF2462 may be essential for *Bacteroides* viability. An interesting alternative comes from the observation that dihydrosphingosine, the putative substrate of BF2462, is toxic to *Bacteroides melaninogenicus* at 4 µM [11]; the absence of BF2462 could therefore lead to the buildup of a toxic intermediate.

**Figure 2:**
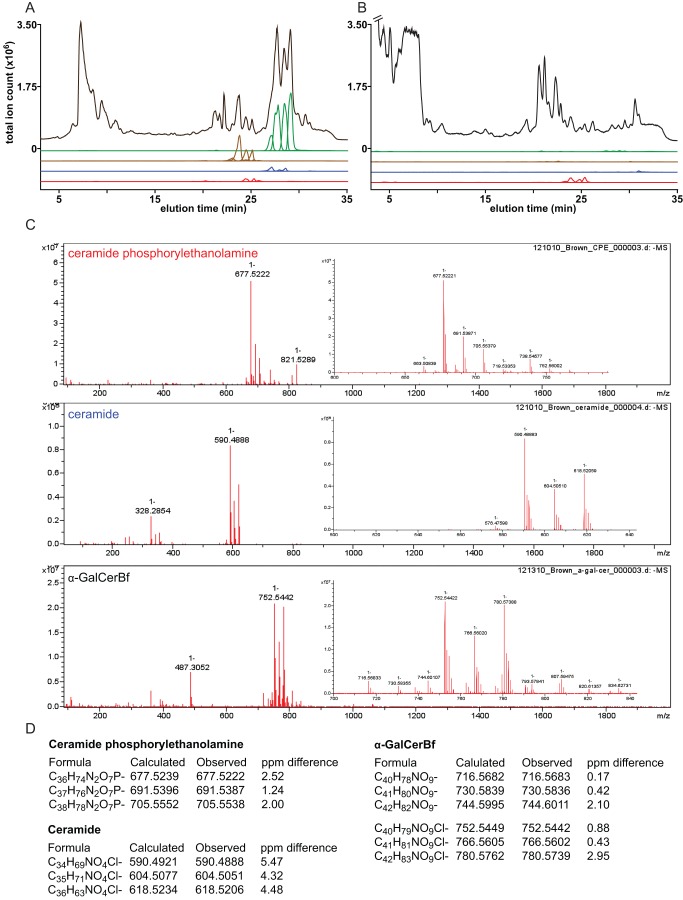
*B.* *fragilis* Δ2461 is deficient in the production of sphingolipids. HPLC-MS traces of crude lipid extracts of (A) wild-type *B. fragilis* and (B) the sphingolipid-deficient mutant ΔBF2461 are shown. The traces shown are the total ion count (black) and the extracted ion traces of sphingolipid masses for ceramide (m/z [M-H]: 540.5, 554.5, 568.5, 582.6; green), CPE (m/z [M-H]: 663.5, 677.5, 691.5, 705.5; brown), α-GalCer_Bf_ (m/z [M-H]: 702.6, 716.6, 730.6, 744.6; blue), and phosphatidylethanolamine (m/z [M-H]: 648.5, 662.5, 676.5, 690.5). Peaks corresponding to the three sphingolipids, but not the phospholipid phosphatidylethanolamine, are absent in *B. fragilis* Δ2461. (C) High-resolution mass spectra of CPE, ceramide_Bf_, and α-GalCer_Bf_ collected in the negative ion mode. The insets show a zoomed-in view of the dominant field of peaks for each compound. (D) A table showing the calculated and observed masses for the dominant mass ions for each compound. See S1.1 in Supporting Information S1 for details.

Nevertheless, since the yeast homolog of BF2461 constitutes the entry point to the sphingolipid pathway, we hypothesized that the Δ2461 mutant would be sphingolipid-deficient, providing an ideal starting point for enumerating the *B. fragilis* sphingolipids. To test our hypothesis, we used comparative HPLC-ELSD to analyze alkaline-stable lipid extracts from the wild-type (WT) and Δ2461 strains. Our analysis revealed three primary peaks that were present in the WT but not the Δ2461 extract (Figure 2). Preparative thin layer chromatography was used to purify multimilligram quantities of these compounds, and HPLC-MS analysis of the purified material revealed that each peak consists of a mixture of co-migrating compounds that vary in mass by 14 Da. Measured in negative mode, the most abundant mass ions for peaks 1, 2, and 3 were 677.5 Da, 554.5 Da, and 716.6 Da, respectively.

### Elucidating the Structures of the *B. fragilis* Sphingolipids

To solve the chemical structures of the sphingolipid species, we first subjected the purified compounds to high-resolution MS. The mass of peak 1 was consistent with ceramide phosphorylethanolamine (CPE) (C_36_H_74_N_2_O_7_P; [M-H]^−^ *m/z*: calculated 677.5234, observed 677.5221), a sphingomyelin isoform previously found to be the principal *B. fragilis* sphingolipid, while the mass of peak 2 was consistent with the corresponding dihydroceramide base (C_34_H_68_NO_4_; [M-H]^−^ *m/z*: calculated 554.5148, observed 554.5156) (Figure 1A; Figure S1 in Supporting Information S1). A set of 1D and 2D NMR experiments on the purified compounds from peaks 1 and 2 yielded resonances and couplings consistent with these assignments (see S4.1 and S4.3 in Supporting Information S1).

### *B. fragilis* Produces α-Galactosylceramide

In contrast, peak 3 was not a known compound. High-resolution MS analysis of the purified material from peak 3 was consistent with an empirical formula of C_40_H_79_NO_9_ ([M-H]^−^ *m/z*: calculated 716.5682, observed 716.5698). 2D NMR analysis indicated that this compound and CPE harbor an identical dihydroceramide base (C_34_H_68_NO_4_), suggesting that the difference (C_6_H_11_O_5_) corresponded to a distinct head group. Four lines of evidence suggest that this head group is an α-configured galactose: (*i*) The molecular formula is consistent with a glycosphingolipid bearing a hexose as a head group. (*ii*) MS/MS analysis reveals a fragment that is consistent with the elimination of a hexose head group from a ceramide base ([M-H]^−^ *m/z*: calculated 536.5048, observed 536.5055). (*iii*) The ^1^H NMR spectrum shows an anomeric proton with a chemical shift of 4.64, consistent with an α-linkage. (*iv*) Chemically synthesized α-galactosylceramide, prepared by selective α-galactosylation of the *B. fragilis* dihydroceramide base (see S1.10 in Supporting Information S1), has a ^1^H NMR spectrum indistinguishable from that of peak 3 (see S4.2 in Supporting Information S1). We term this novel glycosphingolipid *B. fragilis* α-galactosylceramide (α-GalCer_Bf_) (Figure 1B). α-GalCer_Bf_, CPE, and the ceramide base were each purified as an inseparable mixture of varying lipid chain length. This inseparable mixture of alpha-galactosylceramides, hereafter “purified α-GalCer_Bf_,” was the material used for the immunological experiments described below.

α-GalCer_Bf_ is a close structural relative of the sponge-derived α-galactosylceramide agelasphin-9b (Figure 1B) [21]; aside from α-GalCer_Bf_ and the sponge-derived agelasphins, no naturally occurring α-galactosylceramides have ever been discovered. Substantial data have accumulated suggesting that α-GalCer is a ligand for a subset of human and mouse T cells, termed invariant natural killer T cells (iNKT), which express a conserved T cell receptor (TCR) that recognizes glycolipids presented by the major histocompatibility complex class I-like molecule, CD1d [22]. A synthetic derivative of agelasphin-9b termed KRN7000 (Figure 1B) is the prototypical agonist of iNKT cells and has become a critically important reagent for studying NKT cell biology both *in vitro* and *in vivo*. Indeed, iNKT cells are often identified or isolated by flow cytometry on the basis of their ability to bind a synthetic tetramer of CD1d loaded with a derivative of KRN7000. A variety of iNKT cell ligands have been described. One class consists of low-affinity host-derived self-ligands such as isoglobotrihexosylceramide and β-glucopyranosylceramide [23],[24]. Another class includes glycolipids from bacterial species including GSL-1 from *Sphingomonas*, BbGL-II from *Borrelia*, and a family of diacylglycerol-containing glycolipids from *Streptococcus pneumoniae*, all of which have been postulated to be naturally occurring ligands for CD1d [25]–[27]. It has also been proposed that liver infection by *Novosphingobium aromaticivorans*, a close relative of *Sphingomonas* that produces CD1d-binding sphingolipids, results in an NKT-cell-dependent autoimmune response against the liver and bile ducts [28].

### Purified α-GalCer_Bf_ Binds to CD1d and Stimulates Mouse and Human iNKT Cells

**Figure 3:**
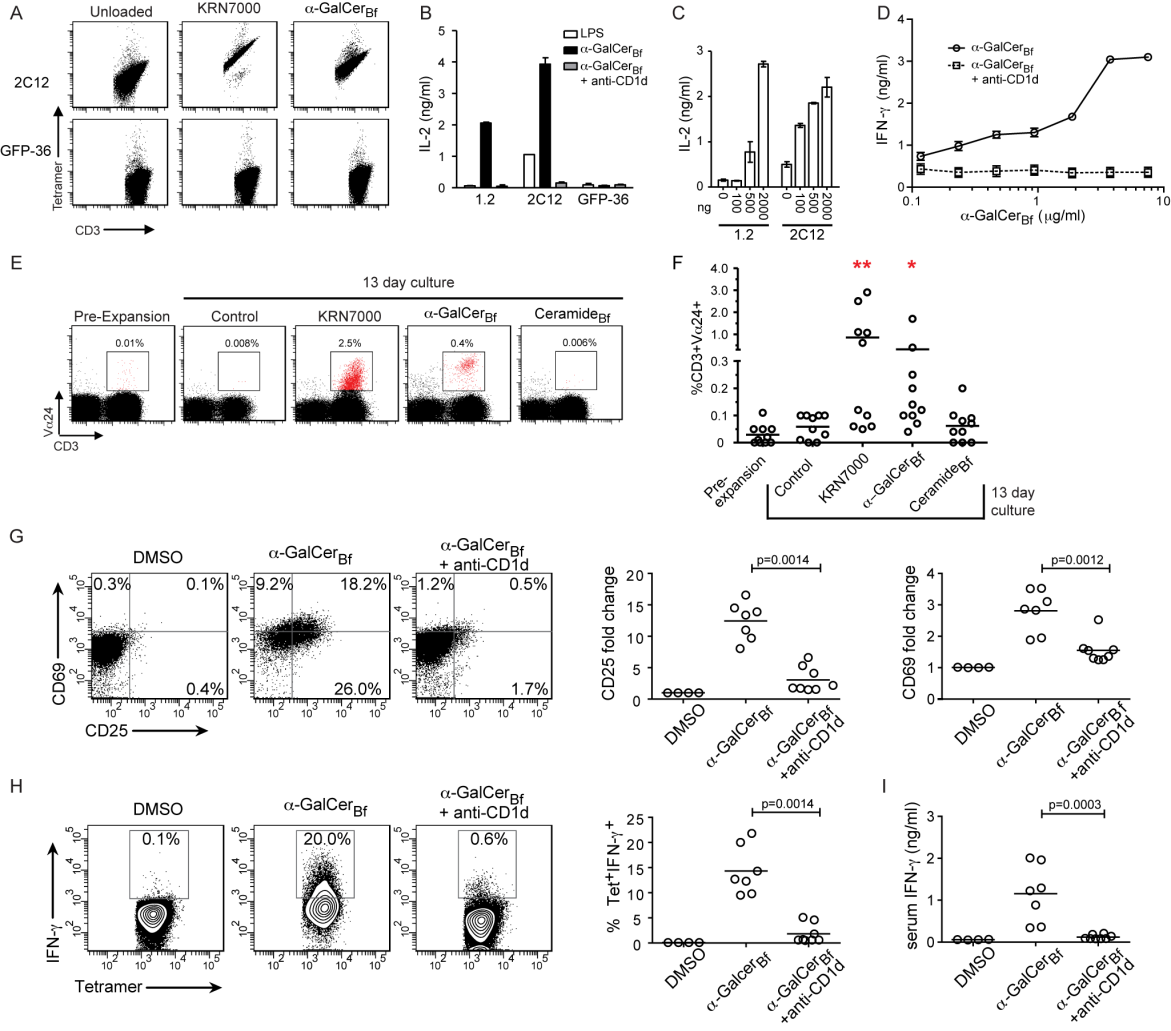
α-GalCer_Bf_ binds CD1d and activates NKT cells. (A) Hybridomas were stained with anti-CD3 antibodies and empty mCD1d tetramers or CD1d tetramers loaded with α-GalCer_Bf_ or KRN7000. Flow cytometry plots are pregated on DAPI^−^ events in lymphocyte gate stained with CD3 antibodies and the specified tetramer. Plots representative of three independent experiments are shown. (B) Hybridomas were cultured with BMDCs pre-pulsed with LPS or LPS + α-GalCer_Bf_ in the presence of control Ig or anti-CD1d blocking antibodies. IL-2 secretion was measured in supernatants 16 h later. Data are representative of three independent experiments. (C) Plates were coated with CD1d monomers and loaded with the specified amounts of α-GalCer_Bf_. Hybridomas were then incubated for 16–18 h and IL-2 was measured in the supernatants by ELISA. Data are representative of three independent experiments. (D) Liver mononuclear cells were cultured with splenocytes plus increasing amounts of α-GalCer_Bf_ in the presence or absence of anti-CD1d blocking antibodies. IFN-γ secretion was measured in supernatants on day 5. Data are representative of three independent experiments. (E and F) Representative flow cytometry plots and pooled data of PBMCs cultured for 13–14 d with 0.1 µg/ml KRN7000, 1 µg/ml α-GalCer_Bf_, or 1 µg/ml ceramide_Bf_. Dot plots show all events in the lymphocyte gate stained with 6B11 (specific for Vα24) and CD3 antibodies. Gate shows percentage of Vα24^+^CD3^+^ NKT cells pre- and postexpansion. Pooled data showing six individual donors tested in three independent experiments. *p = 0.0078, **p = 0.0020 compared to control day 13 culture. (G–I) Bone-marrow-derived dendritic cells were pulsed in vitro with LPS only or LPS + α-GalCer_Bf_ for 24 h. The 0.4×10^6^ cells were transferred to WT mice, which were treated with control Ig or anti-CD1d blocking antibody prior to cell transfer. Liver mononuclear cells were analyzed 16–18 h later. Data shown were pooled from three independent experiments. (G) Expression of CD25 and CD69 on gated CD3^+^tetramer^+^ cells. Representative flow cytometry plots and pooled data showing fold change of CD25 and CD69 surface expression compared to NKT cells isolated from mice transferred with LPS-pulsed BMDCs. (H) Representative flow cytometry plots and pooled data of intracellular IFN-γ expression on gated CD3^+^tetramer^+^ cells. (I) Serum IFN-γ levels.

Based on the striking chemical similarity of α-GalCer_Bf_ to KRN7000, we reasoned that α-GalCer_Bf_ might serve as an endogenous ligand for CD1d and stimulate iNKT cell activity. To test our hypothesis, we began by loading synthetic mouse CD1d tetramers with purified α-GalCer_Bf_ and determining the ability of the sphingolipid/CD1d-tetramer complex (hereafter “tetramer”) to stain two iNKT-cell-derived hybridomas [29],[30]. As with KRN7000, the α-GalCer_Bf_-loaded tetramer (but not an empty tetramer) bound both hybridomas but not a CD4^+^ MHCII restricted hybridoma reactive to GFP (GFP-36) (manuscript in preparation, Yadav and Bluestone), indicating that the tetramer staining was ligand- and TCR-specific (Figure 3A; Figure S2 in Supporting Information S1). The iNKT cell hybridomas tested produced IL-2 in response to both the marine-sponge-derived and *B. fragilis*-derived sphingolipids in a dose-dependent manner and in absence of antigen presenting cells (APCs). These results suggested that α-GalCer_Bf_ is a stimulatory ligand that directly activates iNKT cells *in vitro* (Figure 3B–C; Figure S3 in Supporting Information S1).

We next examined the ability of purified α-GalCer_Bf_ to stimulate freshly isolated mouse and human iNKT cells *in vitro* and *in vivo*. Liver mononuclear cells, 30%–50% of which are NKT cells, were incubated with splenocytes as APCs in the presence of increasing doses of α-GalCer_Bf_ and examined for IFN-γ production. α-GalCer_Bf_ induced IFN-γ in a dose-dependent and CD1d-dependent manner. The response was inhibited completely by anti-CD1d antibodies (Figure 3D), consistent with our previous result that NKT cell stimulation required ligand presentation by CD1d (Figure 3B).

To explore whether the response of NKT cells to α-GalCer_Bf_ is conserved in humans, we determined whether Vα24^+^ cells could be expanded *in vitro* with purified α-GalCer_Bf_ as previously described for KRN7000 [31]. We cultured peripheral blood mononuclear cells (PBMCs) from six independent donors with 0.1 µg/ml KRN7000, 1 µg/ml α-GalCer_Bf_, or 1 µg/ml ceramide_Bf_ for 13 d and assessed the presence of CD3^+^Vα24^+^ cells by flow cytometry (Figure 3E–F). PBMCs cultured with KRN7000 or α-GalCer_Bf_ showed an expansion of a population of CD3^+^Vα24^+^ cells, while PBMCs left untreated or treated with ceramide_Bf_ did not show an expansion of this population. Importantly, this result shows that the activity of α-GalCer_Bf_ is specific and not due to a contaminant of the lipid purification process since ceramide_Bf_, which was purified in a similar manner, did not exhibit this effect. These results demonstrate that α-GalCer_Bf_ has similar activities in murine and human NKT cells and binds human CD1d.

To test whether α-GalCer_Bf_ can activate iNKT cells *in vivo*, mice were immunized with BMDCs pulsed with LPS alone or LPS + purified α-GalCer_Bf_ [32]. Consistent with activation, iNKT cells isolated from the liver showed upregulation of the cell surface markers CD25 and CD69 (Figure 3G), 15% of these liver-resident iNKT cells expressed IFN-γ after treatment (Figure 3H), and elevated IFN-γ levels were observed in the serum of these mice (Figure 3I). Anti-CD1d blocking antibodies inhibited liver iNKT cell activation and IFN-γ production, demonstrating the specificity of iNKT cell activation (Figure 3G–I). We therefore conclude that α-GalCer_Bf_ is capable of stimulating iNKT cell activation and cytokine production *in vivo*.

### A Physiological Context for the Activity of KRN7000

The marine sponge-derived agelasphins and the nonphysiological CD1d ligand KRN7000 have been the basis for numerous studies over the last two decades implicating iNKT cells in immunity (“α-galactosylceramide” has 3,290 citations in Google Scholar, 5/29/12). Unlike the pathogens from which CD1d ligands have previously been isolated, *Bacteroides* is extraordinarily prevalent in the human population, comprising >50% of the trillions of cells in the gut community of a typical human [33]. By showing that *B. fragilis* produces the only known α-galactosylceramide other than the sponge-derived agelasphins, and demonstrating that α-GalCer_Bf_ binds to CD1d and activates iNKT cells *in vitro* and *in vivo*, our results suggest a physiological basis for the activity of KRN7000. It is tempting to speculate that CD1d and iNKT cells function in the context of a microbiota–host interaction, especially in light of a recent report showing that neonatal colonization of germ-free mice by a conventional microbiota downregulates the level of iNKT cells in the colonic lamina propria and lung [34]. Indeed, it has been hypothesized that the agelasphins are not produced by *Agelas mauritianus*, but instead by a bacterial symbiont that inhabits the sponge [22].

In an attempt to determine the *in vivo* effect α-GalCer_Bf_ on NKT cells, we colonized germ-free (GF) mice with WT or sphingolipid-deficient *B. fragilis* by gavage and measured the percent and activation status of NKT cells in the liver and spleen. Colonization was confirmed by fecal cultures and PCR. We varied the length of colonization (1, 3, 4, and 14 d), the mice's age at the time of colonization (4 and 8 wk old), sex, and strain (Swiss Webster and C57BL/6). Several of these experiments indicated an expansion of NKT cells mice colonized by WT but not mutant *B. fragilis*. However, the effect was inconsistent and the levels of NKT cells in our control mice—germ-free (GF) and specific-pathogen-free (SPF)—fluctuated widely. As a percentage of total liver lymphocytes in the GF mice, NKT cells (CD3^+^tetramer^+^) varied between 8% and 48%, making it difficult to draw any conclusions about differences in NKT cell number or activation markers between our experimental data points.

Blumberg and coworkers recently showed that GF mice have increased levels of NKT cells in the colon compared to SPF mice and that colonization of neonatal, but not adult, GF mice with microbiota from SPF mice can reverse this effect [34]. Interestingly, neither the increase nor the reversal after colonization is seen in the liver or the spleen and there were no changes in the activation status of NKT cells. Taken together, our results suggest that the microbiota may affect NKT cells in the colon but not the liver or spleen, and that interventions to change the numbers of NKT cells must occur very early in life and may take weeks to be evident. Although Blumberg and coworkers showed the effects of the microbiota on NKT cell numbers and morbidity in models of IBD and allergic asthma, they did not identify the strain or the molecular pathway responsible for these effects; our results raise the possibility that α-GalCer_Bf_, produced by *B. fragilis*, may be at least partially responsible for the results seen in their models.

There are subtle but important differences between KRN7000 and α-GalCer_Bf_, indicating that the natural ligands for CD1d may be less potent than KRN7000. The principal structural differences between α-GalCer_Bf_ and KRN7000 are (*i*) a shorter *N-*acyl chain bearing a hydroxyl group on the β- rather than the α-carbon, (*ii*) the absence of a hydroxyl group at C4 of the sphinganine base, and (*iii*) iso-branched lipid termini (Figure 1B). Synthetic derivatives of KRN7000 that either have shorter *N-*acyl chains or lack a C4 hydroxyl group have been shown to have less potent activity and/or an altered cytokine response, an effect that might be due to a change in the conformation of the CD1d–lipid complex [35]. Notably, one of the iso-branched lipid termini of α-GalCer_Bf_ is shared with agelasphin 9b. Since iso-branched lipids are commonly associated with specific bacterial genera (for example, comprising 55%–96% of the total fatty acid pool in *Bacteroides*) [36], their presence in agelasphin 9b is consistent with a bacterial origin for these sponge-derived sphingolipids.

### A Proposed Pathway for *Bacteroides* Sphingolipid Biosynthesis

**Figure 4:**
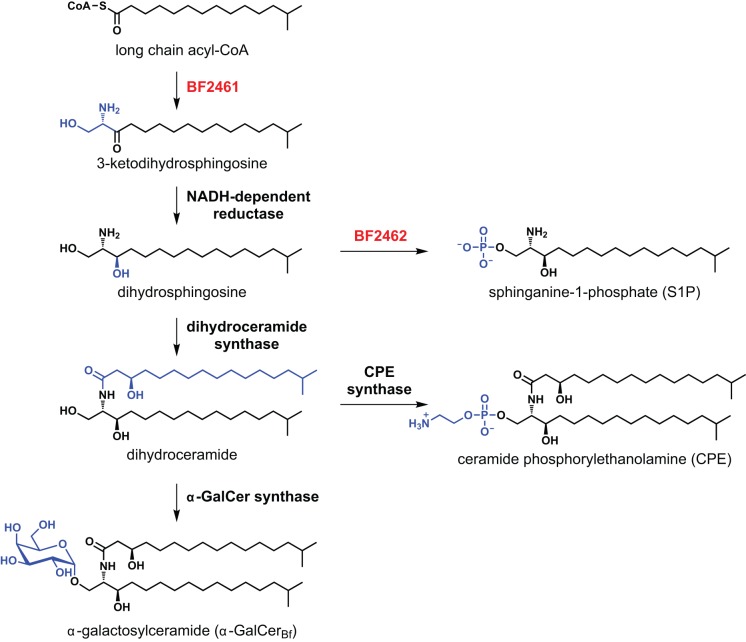
Proposed pathway for *Bacteroides* sphingolipid biosynthesis. BF2461, a putative serine palmitoyltransferase, would catalyze the pyridoxal-phosphate-dependent conjugation of serine and a long-chain acyl-CoA to form 3-ketodihydrosphingosine, which would undergo a ketoreductase-catalyzed conversion to dihydrosphingosine. At this branchpoint, dihydrosphingosine could either be phosphorylated by the putative sphingosine kinase BF2462 to form S1P, or it could undergo *N-*acylation to yield the observed dihydroceramide intermediate (compound 2). This common C_34_ scaffold would then be the substrate for two alternative head group modifications: glycosylation to form α-GalCer_Bf_, or phosphorylethanolamine group transfer to form CPE.

The absence of CPE, dihydroceramide, and α-GalCer_Bf_ from the Δ2461 mutant confirms that BF2461 is involved in *B. fragilis* sphingolipid biosynthesis, marking the first known member of the *Bacteroides* sphingolipid pathway (Figure 4). BF2461 is widely conserved among human-associated genera of Bacteroidales including *Bacteroides*, *Parabacteroides*, *Porphyromonas*, and *Prevotella* (known sphingolipid producers) but absent from *Alistipes* (a nonproducer), supporting its role in the bacterial sphingolipid pathway. Our inability to construct a deletion mutant of BF2462 prevents us from exploring its potential role in the pathway, though it is tempting to speculate that it generates dihydrosphingosine-1-phosphate from dihydrosphingosine. Although the later steps of the pathway remain unclear, the intermediacy of dihydroceramide is supported by the fact that CPE and α-GalCer_Bf_ share a common C_34_ scaffold and by our direct observation of dihydroceramide production by *B. fragilis*. On the basis of these observations, we propose a model of *Bacteroides* sphingolipid biosynthesis that closely mirrors the eukaryotic pathway (Figure 4). Given that sphingolipids comprise ∼30% of total cellular lipids and *Bacteroides* lacks an endoplasmic reticulum (the site of eukaryotic sphingolipid synthesis), the regulation of this pathway in the context of lipid metabolism and the localization of its biosynthetic enzymes will be important areas to explore.

## Materials and Methods

Detailed methods are provided in Supporting Information S1.

### Construction of Mutant Strain ΔBF2461

Primer sequences are listed in Table S1 in Supporting Information S1. DNA fragments flanking BF2461 were PCR amplified from *B. fragilis* NCTC9343 using the following primers: LF_5′; LF_3′; RF_5′; RF_3′. These fragments were digested with SstI and MluI and cloned into the SstI site of pNJR6. The resulting plasmid was introduced into *B. fragilis* NCTC9343 by conjugation, and cointegrates were selected using erythromycin. Cointegrates were passaged, plated on nonselective medium, and replica plated to medium containing erythromycin. Erythromycin-sensitive colonies were screened by PCR to detect those acquiring the mutant genotype.

### Purification of α-GalCer_Bf_

*B. fragilis* NCTC9343 was cultured under standard conditions, and harvested cells were extracted with CHCl_3_∶MeOH (2∶1). The organic extract was subjected to alkaline hydrolysis, neutralized, and extracted with CHCl_3_∶MeOH (2∶1). The crude extract was purified by preparative TLC (CHCl_3_∶MeOH∶H_2_O, 65∶25∶4) to give α-GalCer_Bf_ (R_f_ = 0.6). For complete experimental details, including yields and full characterization (NMR, high-resolution mass spectrometry) of all compounds, see Supporting Information S1. α-GalCer_Bf_ was isolated in five independent batches, and the in vitro and in vivo experiments were repeated with different batches of purified compound.

### α-GalCer_Bf_ Used for Immunological Experiments

α-GalCer_Bf_, CPE, and the ceramide base were each purified as an inseparable mixture of varying lipid chain length. Mass spec analysis of the methanolyzed long chain base (LCB) (S4.6 in Supporting Information S1) suggests that this portion of the structure carries the variation (see next paragraph). The inseparable mixture of alpha-galactosylceramides (>95% pure), referred to as “purified α-GalCer_Bf_,” was the material used for the immunological experiments.

### Analysis of Lipid Tail Length Variation

Methanolysis of ceramide_Bf_ produced a mixture of three LCB amines that could be separated and analyzed by HPLC-MS (S4.6 in Supporting Information S1). Analysis of each by HRMS indicated that they are structural variants that differ in tail chain length. These data suggest the major parent α-GalCer_Bf_ variants (m/z 716.57, m/z 730.58, and m/z 744.60) also differ in chain length of the LCB.

### Hybridoma Stimulation

For dose titration experiments, BMDCs and DN3A4-1.2 and N38-2C12 NKT hybridomas (M. Kronenberg) and GFP36 CD4^+^ hybridoma were cultured at a 3∶1 hybridoma∶BMDC ratio and the indicated doses of KRN7000 or α-GalCer_Bf_ in the presence of 1 µg/ml LPS. Supernatants were harvested after 24 h and IL-2 production was measured by ELISA. For APC-free experiments, CD1d monomers were coated on a 96-well plate for 1 h, and wells were blocked with PBS/10% FBS. The indicated amount of α-GalCer_Bf_ was added to each well and incubated at 37°C for 3 h. After washing unbound α-GalCer_Bf_, hybridomas were added. Supernatants were harvested after 16–18 h and IL-2 production was measured by ELISA. For *in vitro* CD1d blocking experiments, α-GalCer_Bf_ pulsed BMDCs were cultured at a 3∶1 hybridoma∶BMDC ratio in the presence of 10 µg/mL anti-CD1d antibody (Clone 1B1, BD Pharmingen). Supernatants were harvested after 16–18 h and IL-2 production was measured by ELISA.

### *In Vitro* Stimulation of Human NKT Cells

For blood draws from healthy donors, informed consent was obtained in accordance with approved University of California, San Francisco IRB policies and procedures (IRB 10-02596). PBMCs were cultured for 13–14 d in RPMI containing 10% autologous serum plus lipids as described in Figure 3. On day 1 of culture, 100 U/ml hIL-2 was added. Cultures were harvested on day 13 or 14 and the percentage of CD3^+^Vα24^+^ NKT cells was determined by flow cytometry after staining with CD3 and 6B11 antibodies.

### *In Vivo* Activation of NKT Cells

Mice were sacrificed 16–18 h after transfer of 0.4×10^6^ mature CD86^hi^MHCII^hi^ BMDCs. Livers were cut into small pieces and passed through a stainless mesh. Cells were resuspended in 40% Percoll solution (GE Healthcare), underlaid with 60% Percoll solution, and centrifuged at 2,300 rpm for 20 min at room temperature. All isolations were performed in the presence of brefeldin A (Sigma). After cell surface staining, cells were fixed in Cytofix/Cytoperm (BD Biosciences) according to the manufacturer's instructions and stained for intracellular cytokines. Serum IFN-γ was measured by ELISA.

## Supporting Information

Supporting Information S1 **Contents:**
*Section S1*, materials, equipment, and general methods.
*Section S2*, high-resolution mass spectrometry and LC-MS analysis.
*Section S3*, in vitro titration data.
*Section S4*, spectral data.
*Figure S1*, *B. fragilis* Δ2461 is deficient in the production of sphingolipids. LC-MS trace with extracted ions shown [M-H]. See Figure 2 legend for details.
*Figure S2*, α-GalCer_Bf_ binds CD1d in vitro. Hybridomas were stained with anti-CD3 antibodies and empty mCD1d tetramers or mCD1d tetramers loaded with α-GalCerBf or KRN7000. Flow cytometry plots representative of three independent experiments are shown. (A) Plots show forward (FSC) and side (SSC) scatter of all events. (B) Plots pre-gated as shown in (A) and further gated on DAPI-negative events show staining with tetramer and CD3 antibodies.
*Figure S3*, KRN7000 and α-GalCer_Bf_ titration in vitro. BMDCs and NKT hybridomas were cultured at a 3∶1 hybridoma:BMDC ratio and the indicated doses of KRN7000 or α-GalCer_Bf_ in the presence of 1 µg/ml LPS. Supernatants were harvested after 24 h and IL-2 production was measured by ELISA.
*Table S1*, Primers used in this study.
(PDF)

## Abbreviations

α-GalCer: α-galactosylceramide
APC: antigen presenting cell
CPE: ceramide phosphorylethanolamine
ELSD: evaporative light scattering detector
FBS: fetal bovine serum
GF: germ-free
HPLC: high-performance liquid chromatography
HRMS: high-resolution mass spectrometry
iNKT: cell invariant natural killer T cell
LCB: long-chain base
PBMC: peripheral blood mononuclear cell
PBS: phosphate buffered saline
S1P: sphingosine-1-phosphate
SPF: specific-pathogen-free
TCR: T cell receptor
