# Supplementary material for: Production of α-Galactosylceramide by a Prominent Member of the Human Gut Microbiota
Source: PLoS Biol. 2013 Jul 16;11(7):e1001610. doi: 10.1371/journal.pbio.1001610 (PMC3712910; doi:10.1371/journal.pbio.1001610)

Production of α-galactosylceramide by a prominent member of the human gut microbiota

Laura C. Wieland Brown[1,2]*, Cristina Penaranda[3]*, Purna C. Kashyap[4], Brianna B. Williams[1], Jon Clardy[2], Mitchell Kronenberg[5], Justin L. Sonnenburg[4], Laurie E. Comstock[6], Jeffrey A. Bluestone[3†] & Michael A. Fischbach[1†]

[1]Department of Bioengineering and Therapeutic Sciences and the California Institute for Quantitative Biosciences, University of California, San Francisco, San Francisco, California 94158, USA
[2]Department of Biological Chemistry and Molecular Pharmacology, Harvard Medical School, Boston, Massachusetts 02115, USA
[3]Diabetes Center and the Department of Medicine, University of California, San Francisco, San Francisco, California 94143, USA
[4]Department of Microbiology and Immunology, Stanford University School of Medicine, Stanford, CA 94305, USA
[5]La Jolla Institute for Allergy and Immunology, La Jolla, CA 92037, USA
[6]Channing Laboratory, Department of Medicine, Brigham and Women's Hospital, Harvard Medical School, Boston, Massachusetts 02115, USA

*Denotes equal contribution
[†]Correspondence: fischbach@fischbachgroup.org, jeffreya.bluestone@ucsf.edu

SUPPLEMENTARY INFORMATION

S0. Contents

- S1. Materials, Equipment and General Methods
- S2. High Resolution Mass Spectrometry and LC-MS Analysis
- S3. In vitro Titration
- S4. Spectral Data

S1. Materials and Methods

S1.1. Materials, general methods, and instrumentation used in sphingolipid isolation and purification. All solvents were purchased from Sigma–Aldrich unless otherwise noted. Low-resolution LC-MS analysis was carried out on an Agilent 6130 LC/MS using a Phenomenex Gemini-NX 5 µm C18 110 Å 100 x 2 mm column at 0.7 ml/min. LC-MS data was collected in negative ion mode, on an Agilent 6520 Accurate-Mass Q-TOF Mass Spectrometer fitted with an electrospray ionization (ESI) source. The capillary voltage was set to 3500 kV, and the fragmentor voltage at 125 V. The drying gas temperature was maintained at 320 °C with a flow rate of 12 L/min and a nebulizer pressure of 45 psi. HPLC separation was effected on a Gemini-NX C18 reverse phase column (5µm, 110 Å, 2.0 x 50 mm, Phenomonex). Compounds were eluted at 0.5 ml/min in a gradient of solvents

A (0.1% NH_4OH in water) and B (0.1% NH_4OH in methanol): 65% B increasing to 100% B over 30 min., isocratic at 100% B for 1 min. before returning to 65%B and re-equilibrating over 3 min. High resolution mass spectrometry (HRMS) was carried out by Ted Voss at the WM Keck Foundation Biotechnology Resource Laboratory at Yale University on a Bruker 9.4T FT-ICR MS. Infrared (IR) spectra are recorded on a Perkin Elmer 781 spectrophotometer, λ_{max} in cm^{-1}. Bands are characterized as broad (br), strong (s), medium (m) or weak (w). ^1H NMR spectra were recorded on a Varian Unity 600 MHz spectrometer. Chemical shifts are reported in ppm from tetramethylsilane with the solvent resonance resulting from incomplete deuteration as the internal standard ($CDCl_3$: δ 7.26, CD_3OD: δ 3.31, DMSO: δ 2.50, pyridine: δ 8.74). Data are reported in table form as follows: chemical shift, multiplicity (s = singlet, d = doublet, t = triplet, q = quartet, br = broad, m = multiplet), coupling constants, and integration. ^{13}C NMR spectra were recorded on a Varian Unity 400 MHz spectrometer (100 MHz) with complete proton decoupling. Chemical shifts are reported in ppm from tetramethylsilane with the solvent resonance as the internal standard ($CDCl_3$: δ 77.16, CD_3OD: δ 49.00, DMSO: δ 39.52). Optical rotations were measured on a Perkin Elmer 241 Polarimeter. Unless otherwise noted, all solvents and reagents were purchased from VWR or Fisher and used without further purification. An anaerobic environment was achieved by three methods, and all were equally effective and used interchangeably: 1) Coy Laboratory Products anaerobic chamber; 2) BD GasPak™ EZ Gas Generating Container System; 3) Mitsubishi Gas Chemical Co. AnaeroPack™ System.

S1.2. Reagents used in immunology studies. PBS-57 loaded mCD1d tetramers, which were used for mouse experiments, and unloaded mCD1d monomers were obtained from the NIH Tetramer Core Facility. For loading, monomers were incubated with KRN7000 or α-$GalCer_{Bf}$ diluted in DMSO at 6 fold molar excess at 37°C for 3 hrs in the presence of pepstatin, leupeptin, EDTA and Tween20. Samples were concentrated using a 30K microconcentrator. Monomers were tetramerized by incubation with strepavidin-conjugated APC (Molecular probes) at a 1:1 ratio. Staining was performed at room temperature for 1hr. CD4 (RM4-5), CD3 (2C11), CD69 (H1.2F3), CD25 (PC61), NK1.1 (PK136), CD86 (GL1), MHCII (NIMR-4), IFNγ (XMG1.2) were purchased (BioLegend, eBiosciences, SBA or Pharmingen). For cell surface staining, cells were preincubated with 2.4G2 culture supernatant (UCSF cell culture facility) and incubated with antibodies/tetramer for 30 minutes at 4 °C. Viability was determined by staining cells with LIVE/DEAD fixable Aqua stain (Invitrogen) per manufacturer's instructions. 200ug Anti-CD1d antibody (1B1 clone, eBiosciences) was administered i.v. 5-30 minutes prior to BMDC transfer. Rat IgG2b (LTF-2, UCSF cell culture facility) was used as isotype control. KRN7000 was purchased from Avanti Polar Lipids.

S1.3. Bacterial Strains and Growth Conditions. *B. fragilis* NCTC 9343 was the type strain used in this study and was the parental strain of the deletion mutants. *Bacteroides* were grown anaerobically in basal medium or on brain-heart infusion plates supplemented with hemin (50 μg/ml) and vitamin K_1 (0.5 μg/ml) (BHIS plates), with gentamicin (200 μg/ml) and erythromycin (5 μg/ml) added where appropriate. *E. coli* DH5α containing recombinant plasmids was grown in L broth or on L agar plates containing kanamycin (50 μg/ml).

S1.4. Mice. For *in vivo* activation of NKT cells, C57BL/6 female mice aged 6-12 weeks purchased from Jackson Laboratory (Bar Harbor, ME) were used. Mice were housed under specific pathogen-free conditions at the University of California, San Francisco Animal Barrier Facility and experiments were approved by the Institutional Animal Care and Use Committee of the University of California, San Francisco.

S1.5. Bone Marrow-Derived Dendritic Cells. Bone marrow progenitors were cultured in IMDM containing 10% FBS with addition of 20 ng/ml GM-CSF (G6 supernatant, Abbas Laboratory) starting on day 2 and 1 ng/ml IL-4 (I3L6 supernatant, Abbas Laboratory) on day 6. For in vivo transfers and in vitro CD1d blocking experiments, BMDCs were pulsed with 1ng/mL LPS (*Escherichia coli* O26:B6; Sigma-Aldrich) +/- 10 ug/mL α-GalCer$_{Bf}$ on day 8. After overnight culture, cells were harvested and washed twice before use.

S1.6. Statistical analysis. The statistical significance of differences in Fig. 3f between each group and the "nothing" control was determined by the Wilcoxon matched-pairs signed rank test using Prism software. The statistical significance of differences between groups in Fig. 4g was determined by the Mann-Whitney test using Prism software.

S1.7. Construction of Δ***BF2461 deletion mutant.*** Creation of the deletion mutant involved PCR amplification of DNA flanking the each side of the region to be deleted, digestion of these products with restriction enzymes utilizing sites engineered into the primers (underlined below), and three-way ligation into *Sst*I or *Bam*HI site of the *Bacteroides* conjugal suicide vector pNJR6. The resulting plasmid in *E. coli* DH5α was conjugally transferred into *B. fragilis* and cointegrates were selected by Em^r. Cointegrates were passaged, plated on nonselective medium, and replica plated to medium containing erythromycin. Em^s colonies were screened by PCR to detect those acquiring the mutant genotype. The BF2461 deletion mutant was constructed so that 1,078 bp of the 1,185-bp gene was deleted.

Table S1. Primers used in this study

Primer	Sequence	Comments
**2461_LF_5'**	5′-CCTTGAGCTCCAGTTCGATATTACGGATCACCTT-3′	ΔBF2461 - left flanking region - 5'
**2461_LF_3'**	5′-CTGCACGCGTTATACGCCTTTAGCCTTTATCTGC-3′	ΔBF2461 - left flanking region - 3'
**2461_RF_5'**	5′-GGCAACGCGTAAGTTAGTGAAATGTTTCAAGGCA-3′	ΔBF2461 - right flanking region - 5'
**2461_RF_3'**	5′-TTTGGAGCTCTTCAATAGTGTAGGAAGCGTTTTG-3′	ΔBF2461 - right flanking region - 3'

S1.8. Growth Measurements. *B. fragilis* NCTC 9343 and mutant strains were cultured by inoculating 100 mL basal medium with growth from a freshly inoculated BHIS plate to an OD_{600} of 0.01. OD600 was recorded at regular intervals and CFU/mL was determined at each time point.

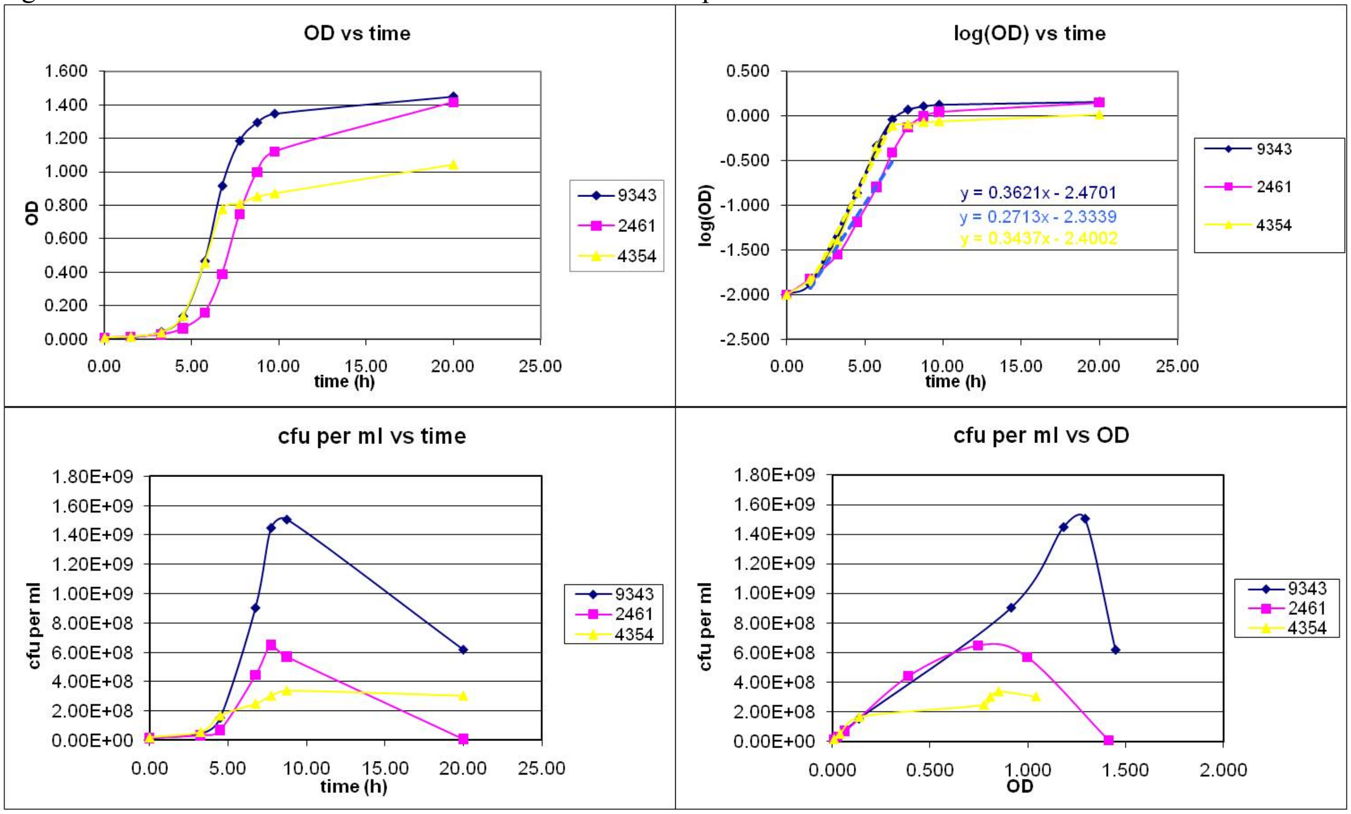

S1.9. Purification of sphingolipids and free ceramide from* B. fragilis *NCTC 9343. *B. fragilis* NCTC 9343 was allowed to grow under an anaerobic atmosphere in basal medium (4.5 L) supplemented with hemin (50 µg/ml) and vitamin K_1 (0.5 µg/ml) at 37 ºC for 2d. The cells were harvested by centrifugation and extracted with $CHCl_3$:MeOH (2:1, 1.5 L). The organic extract was filtered and concentrated, then re-dissolved in $CHCl_3$:MeOH (2:1, 100 ml) and treated with NaOH (0.5 N, 100 ml). The suspension was allowed to stir at 37 ºC for 1 h, then brought to pH 2-4 with 10% HCl. The aqueous layer was extracted with $CHCl_3$:MeOH (2:1, 3 x 500 ml). The organic layers were combined, dried (Na_2SO_4), and concentrated to give 0.5 g crude extract. The crude extract was dissolved in a minimum amount of $CHCl_3$:MeOH (2:1), applied to a 2 mm preparative TLC plate and eluted with $CHCl_3$:MeOH:AcOH:H_2O (100:20:12:5). The plate was divided into three section based on polarity (top,

middle, and bottom). Each section was scraped off the plate and extracted with $CHCl_3$:MeOH (5:1). The resulting solution was concentrated and further purified by preparative TLC (0.5 mm): the top section was eluted in $CHCl_3$:MeOH:NH_4OH (95:5:0.8) to give purified **ceramide**[1] (11.4 mg, white solid, Rf = 0.3). The middle section was eluted in $CHCl_3$:MeOH:H_2O (first in 88:12:0.5, then in 65:25:4) to give α-galactosylceramide (**α-GalCer$_{Bf}$**; 2.7 mg, glass, Rf = 0.6 in 65:24:4). The bottom section was eluted in $CHCl_3$:MeOH:AcOH:H_2O (100:20:12:5) to give purified ceramide phosphorylethanolamine (**CPE**; 4.2 mg, white solid, Rf = 0.2). Each compound was isolated as a mixture of compounds with varying lipid chain lengths and was not further separated. **α-GalCer$_{Bf}$** comprises ~0.5% of the extractable lipids as determined by HPLC/MS.

Ceramide: **IR** (neat): 3296.1 (br, s), 2917.8 (s), 2849.3 (s), 1639.3 (m), 1547.6 (m), 1467.1 (m), 1420.1 (w), 1383.0 (w), 1365.7 (w), 1249.8 (w), 1108.9 (w), 1021.9 (w). **^{1}H NMR (600 MHz, 2:1 $CDCl_3$:CD_3OD)**: δ 7.37 (d, *J* = 8.6 Hz, 1H), 3.94 – 3.89 (m, 1H), 3.81 – 3.76 (m, 1H), 3.73 (dd, *J* = 11.5, 5.3 Hz, 1H), 3.66 (dd, *J* = 11.5, 3.7 Hz, 1H), 3.63 – 3.55 (m, 1H), 2.35 (dd, *J* = 14.5, 3.3 Hz, 1H), 2.25 (dd, *J* = 14.6, 8.9 Hz, 1H), 1.52 – 1.34 (m, 6H), 1.31 – 1.18 (m, 36H), 1.14 – 1.02 (m, 4H), 0.84 – 0.77 (m, 12H). **^{13}C NMR (100 MHz, 2:1 $CDCl_3$:CD_3OD):** δ 173.04, 72.23, 68.57, 61.22, 54.68, 43.34, 38.95, 37.05, 36.52, 34.30, 33.88, 31.80, 29.90, 29.81, 29.59, 29.56, 29.55, 29.52, 29.51, 29.48, 29.36, 29.23, 27.84, 27.29, 26.98, 25.79, 25.40, 22.53, 22.36, 18.94, 13.78, 11.09. For spectra, see page S9. **Optical rotation:** $[\alpha]_D^{22}$ -1.73 (*c* = 0.86, 2:1 $CHCl_3$:MeOH). **HRMS** *m/z* calcd for $C_{34}H_{68}NO_4^-$ $(M-H)^-$: 554.51538. Found: 554.51560 $(M-H)^-$. **HRMS** *m/z* calcd for $C_{35}H_{70}NO_4^-$ $(M-H)^-$: 568.53103. Found: 568.53117 $(M-H)^-$.

α-GalCer$_{Bf}$: IR (neat): 3278.6 (br, s), 2919.4 (s), 2850.5 (s), 1643.8 (m), 1620.8 (m), 1563.1 (m), 1465.5 (m), 1365.4 (w), 1342.3 (w), 1024.8 (s). **^{1}H NMR (600 MHz, DMSO)**: δ 7.60 (d, *J* = 9.1 Hz, 1H), 4.64 (d, *J* = 3.3 Hz, 1H), 4.58 (d, *J* = 4.9 Hz, 1H), 4.52 (d, *J* = 6.4 Hz, 1H), 4.49 (d, *J* = 5.6 Hz, 1H), 4.47 (d, *J* = 5.6 Hz, 1H),4.33 (d, *J* = 4.2 Hz, 1H), 4.15 (d, *J* = 7.6 Hz, 1H), 3.80 – 3.74 (m, 1H), 3.74 – 3.69 (m, 1H), 3.69 – 3.64 (m, 1H), 3.60 – 3.36 (m, 7H), 2.17 (ddd, *J* = 26.3, 13.8, 6.6 Hz, 2H), 1.52 – 1.43 (m, 3H), 1.43 – 1.26 (m, 2H), 1.21 (br s, 37H), 1.14 – 1.06 (m, 4H), 0.82 (d, *J* = 6.6 Hz, 12H). **^{13}C NMR (100 MHz, DMSO):** δ 170.79, 99.95, 71.64, 70.15, 69.61, 69.32, 69.13, 67.90, 67.36, 61.02, 53.39, 44.47, 38.92, 36.99, 34.19, 34.07, 31.74, 27.85, 27.24, 26.91, 25.56, 22.91. For spectra, see page S16. **Optical rotation:** $[\alpha]_D^{22}$ +60.47 (*c* = 0.13, 2:1 $CHCl_3$:MeOH). **HRMS** *m/z* calcd for $C_{40}H_{78}NO_9^-$ $(M-H)^-$: 716.56821. Found: 716.56860 $(M-H)^-$. **HRMS** *m/z* calcd for $C_{41}H_{80}NO_9^-$ (M-H): 730.58386. Found: 730.58393 $(M-H)^-$.

CPE: **IR** (neat): 3322.0 (br, s), 2918.3 (s), 2849.6 (s), 1649.9 (s), 1559.3 (s), 1466.2 (m), 1466.2 (m), 1410.0 (m), 1221.8 (s), 1080.4 (s), 1021.1 (s). **^{1}H NMR (600 MHz, 2:1 $CDCl_3$:CD_3OD):** δ 4.11 – 4.05 (m, 1H), 4.04 – 3.94 (m, 2H), 3.94 – 3.83 (m, 3H), 3.56 (t, *J* = 7.9 Hz, 1H), 3.09 – 2.99 (m, 2H), 2.34 (dd, *J* = 14.6, 3.3 Hz, 1H), 2.25 (dd, *J* = 14.6, 9.3 Hz, 1H), 1.54 – 1.31 (m, 6H), 1.22 (s, 36H), 1.15 – 1.11 (m, 4H), 0.82 (d, *J* = 6.6 Hz, 12H). **^{13}C NMR (100 MHz, 2:1 $CDCl_3$:CD_3OD)** δ 172.65, 69.69, 68.33, 64.26, 61.46, 53.95, 53.89, 43.28, 40.23, 40.16, 38.76, 37.01, 33.31, 31.61, 29.61, 29.38, 29.15, 29.03, 27.64, 27.09, 25.53, 25.28, 22.04. For spectra, see page S25. **Optical rotation:** $[\alpha]_D^{22}$ +13.30 (*c* = 0.28, 2:1 $CHCl_3$:MeOH). **HRMS** *m/z* calcd for $C_{36}H_{74}N_2O_7P^-$ $(M-H)^-$: 677.52391. Found: 677.52210 $(M-H)^-$. **HRMS** *m/z* calcd for $C_{37}H_{76}N_2O_7P^-$ $(M-H)^-$: 691.53956. Found: 691.53860 $(M-H)^-$. **HRMS** *m/z* calcd for $C_{38}H_{78}N_2O_7P^-$ $(M-H)^-$: 705.55521. Found: 705.55373 $(M-H)^-$.

1. Miyagawa, E., Azuma, R., Suto, T. Distribution of Sphingolipids in *Bacteroides* Species. *J. Gen. Appl. Microbiol.* **24,** 341-348 (1978).

S1.10. Semi-synthetic generation of α-GalCer$_{Bf}$.

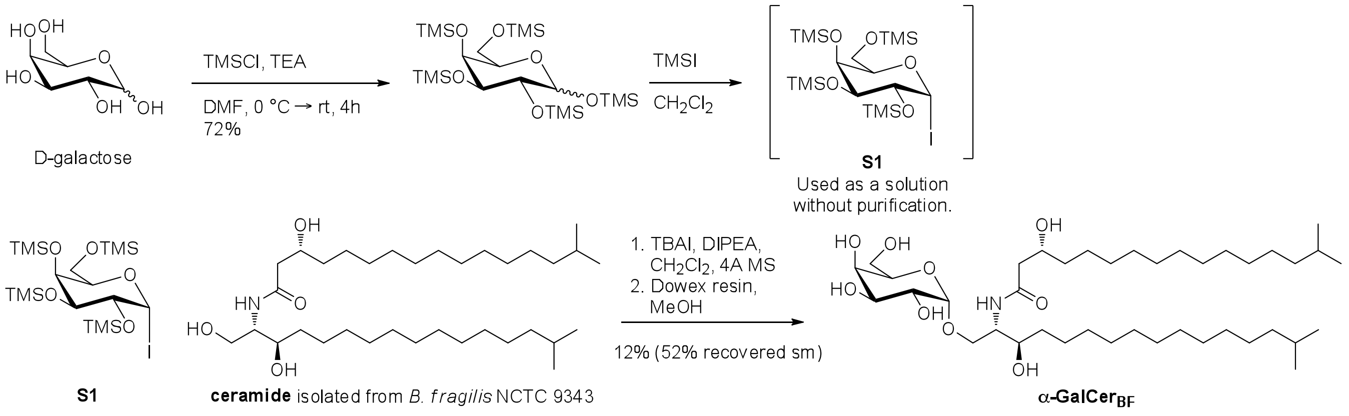

TMS-protected galactose[2] and α-iodo-substituted compound **S1**[3] were prepared as previously described. Ceramide (5.0 mg, 0.0088 mmol) was isolated from *B. fragilis* NCTC 9343 as described above. α-Selective glycosylation was carried out as previously described, and α-GalCer$_{Bf}$ was obtained after purification by preparative TLC as described above in 12% yield (0.74 mg, 0.0010 mmol). Unreacted ceramide was recovered in 52% after purification (2.6 mg, 0.0046 mmol). ^{1}H NMR analysis (600 MHz, DMSO) of semisynthetic α-GalCer$_{Bf}$ indicated that it was identical to α-GalCer$_{Bf}$ isolated from *B. fragilis* NCTC 9343.

^{1}H NMR (600 MHz, DMSO) δ 7.60 (d, *J* = 9.4 Hz, 1H), 4.64 (d, *J* = 3.2 Hz, 1H), 4.57 (d, *J* = 4.9 Hz, 1H), 4.52 (d, *J* = 6.5 Hz, 1H), 4.49 (d, *J* = 5.6 Hz, 1H), 4.47 (d, *J* = 5.6 Hz, 1H), 4.32 (d, *J* = 4.4 Hz, 1H), 4.14 (d, *J* = 8.2 Hz, 1H), 3.78 – 3.74 (m, 1H), 3.74 – 3.69 (m, 1H), 3.68 – 3.64 (m, 1H), 3.61 – 3.36 (m, 7H), 2.17 (ddd, *J* = 20.4, 13.7, 6.9 Hz, 2H), 1.51 – 1.43 (m, 3H), 1.42 – 1.26 (m, 2H), 1.21 (s, 37H), 1.13 – 1.09 (m, 4H), 0.82 (d, *J* = 6.6 Hz, 12H).

---

2. Bhat, A. S., Gervay-Hague, J. Efficient Syntheses of b-Cyanosugars Using Glycosyl Iodides Derived from Per-*O*-silylated Mono- and Disaccharides. *Org. Lett.* **3**, 2081-2084 (2001).
3. Schombs, M., Park, F. E., Du, W., Kulkarni, S. S., Gervay-Hague, J. One-Pot Syntheses of Immunostimulatory Glycolipids. *J. Org. Chem.* **75**, 4891-4898 (2010).

S1.11. Methanolysis of ceramide and determination of absolute configuration.

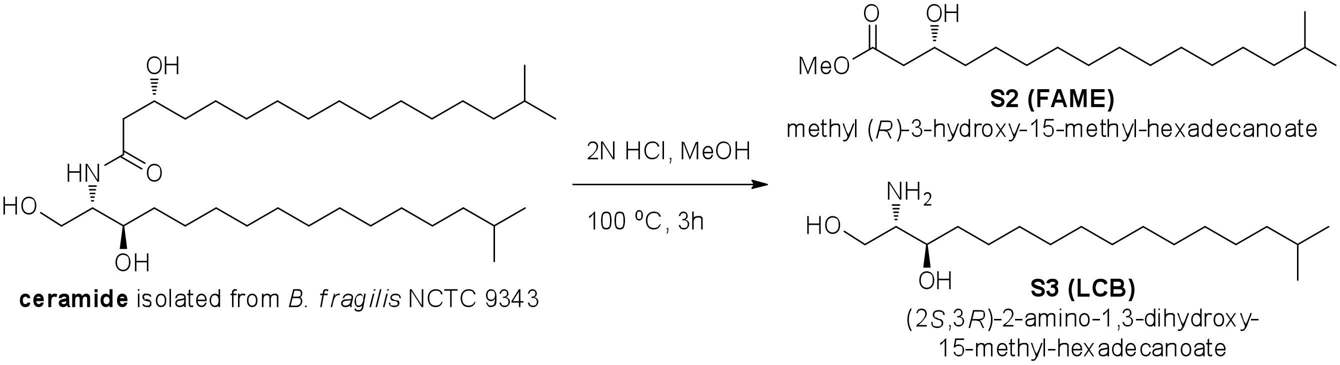

Ceramide isolated from *B. fragilis* NCTC 9343 (10 mg, 0.018 mmol) was suspended in a solution of HCl in methanol (2 mL, 2N) in a sealed tube.[4] The mixture was kept at 100 ºC for 3 h, then allowed to cool to room temperature. Water (1mL) was added, after which the aqueous layer was washed with hexanes (3 x 3 mL). The aqueous layer was set aside. The organic layers were combined, dried over Na_2SO_4, and the volatiles were removed *in vacuo*. The resulting residue, which contained fatty acide methyl ester (FAME) **S2**, was purified by silica gel chromatography (10:1 hexanes:EtOAc) to furnish pure **S2** (2.8 mg, 0.0092 mmol, 51%). The aqueous layer was treated with concentrated KOH until a pH of 10-12 was reached, then washed with Et_2O (2 x 2 mL). The organic layers were combined, dried over Na_2SO_4, and the volatiles were removed *in vacuo*. The resulting residue, which contained long chain base (LCB) **S3**, was purified by preparative TLC (silica gel, 100:20:12:5 $CHCl_3$:MeOH:AcOH:H_2O, R_f = 0.37) to furnish pure **S3** (1.1 mg, 0.0038 mmol, 21%).

S2 (FAME): **IR** (neat): 2922.17 (s), 2852.1 (s), 1725.3 (s), 1463.6 (w), 1436.8 (m), 1365.1 (w), 1168.5 (m), 1051.9 (m). **^{1}H NMR (600 MHz, cdcl$_3$)** δ 4.00 (dtd, *J* = 12.2, 4.4, 3.1 Hz, 1H), 3.71 (s, 3H), 2.51 (dd, *J* = 16.4, 3.0 Hz, 1H), 2.41 (dd, *J* = 16.4, 9.1 Hz, 1H), 1.60 – 1.48 (m, 2H), 1.47 – 1.38 (m, 2H), 1.38 – 1.19 (m, 18H), 1.15 (dd, *J* = 14.1, 6.7 Hz, 2H), 0.86 (d, *J* = 6.6 Hz, 6H). **HRMS** Calcd for $C_{18}H_{37}O_3$ [M + H]: 301.273721; Found [M + H]: 301.27323. **Optical rotation:** $[\alpha]_D^{22}$ -14.8 (*c* = 0.183, $CHCl_3$). The absolute configuration was assigned based on comparison with a reported value.[5]

S3 (LCB): **IR** (neat): 3373.2 (br, s), 2921.2 (s), 2851.7 (s), 1631.9 (m), 1588.7 (m), 1548.7 (w), 1510.0 (w), 1464.9 (w), 1367.5 (m), 1144.9 (w), 1050.8 (w). **^{1}H NMR (600 MHz, pyridine)** δ 4.29 (dd, *J* = 10.4, 4.4 Hz, 1H), 4.11 (dd, *J* = 10.4, 7.3 Hz, 1H), 4.06 – 3.99 (m, 1H), 3.36 – 3.26 (m, 1H), 1.92 – 1.77 (m, 3H), 1.65 – 1.52 (m, 1H), 1.53 – 1.34 (m, 4H), 1.27 (s, 17H), 1.18 – 1.10 (m, 2H), 0.87 (d, *J* = 6.6 Hz, 6H). **HRMS** Calcd for $C_{17}H_{38}NO_2^+$ $[M + H]^+$: 288.289706; Found $[M + H]^+$: 288.28930; **HRMS** Calcd for $C_{18}H_{40}NO_2^+$ $[M + H]^+$: 302.30590; Found $[M + H]^+$: 302.30480; **HRMS** Calcd for $C_{19}H_{42}NO_2^+$ $[M + H]^+$: 316.32155; Found $[M + H]^+$: 316.31980. **Optical rotation:** $[\alpha]_D^{22}$ +6.0 (*c* = 0.0050, pyr-d5). The absolute configuration was assigned based on comparison with reported values.[6]

---

4. Ceramide isolated from *B. fragilis* NCTC 9343 was methanolyzed according to the procedure described in: Miyagawa, E., Azuma, R., Suto, T., Yano, I. Occurrence of Free Ceramides in *Bacteroides fragilis* NCTC 9343. *J. Biochem.* **86**, 311-320 (1979).
5. Reported values for the optical rotation of methyl (*R*)-3-hydroxy-15-methyl-hexadecanoate: (a) $[\alpha]_D^{25}$ -14.3 (*c* = 0.51, $CHCl_3$):Labeeuw, O., Phansavath, P., Genêt, J.-P. Total synthesis of sulfobacin A through dynamic kinetic resolution of a racemic β-keto-α-amino ester hydrochloride. *Tetranedron: Asymmetry,* **15**, 1899-1908 (2004). (b) $[\alpha]_D^{25}$ -12.7 (*c* = 0.518, $CHCl_3$): Kamiyama, T. *et al.* Sulfobacins A and B, Novel von Willebrand Factor Receptor Antagonists II. Structural Elucidation. *J. Antibiot.* **48**, 929-936 (1995).
6. So, R. C. *et al.* Straightforward Synthesis of Sphinganines via a Serine-derived Weinreb Amide. *J. Org. Chem.* **69,** 3233-3235 (2004).

S2. High Resolution Mass Spectrometry and LC-MS Analysis

S2.1. High Resolution Mass Spectrometry (HRMS).

Table S2. Summary of HRMS data

**Compound**	**source**	**calculated [M-H]**	**observed [M-H]**	**MS data source[a]**	**ppm difference**
**α-GalCer$_{Bf}$**	*B. fragilis* NCTC 9343	716.56821	716.56860	Yale	-0.5
		730.58331	730.58393	Yale	-0.8
**α-GalCer$_{Bf}$**	OMV from	716.56821	716.57000	Yale	-2.5
	B. fragilis NCTC 9343	730.58331	730.58457	Yale	-1.7
		744.59951	744.60133	Yale	-2.4
**α-GalCer$_{Bf}$**	*B. fragilis* 63812	744.59951	744.60120	Yale	-2.3
**ceramide**	*B. fragilis* NCTC 9343	554.51538	554.51560	Yale	-0.4
		568.53103	568.53117	Yale	-0.2
**CPE**	*B. fragilis* NCTC 9343	677.52391	677.52210	Yale	2.7
		691.53956	691.53860	Yale	1.4
		705.55521	705.55373	Yale	2.1
**CPE**	OMV from *B. fragilis* NCTC 9343	691.53956	691.54180	Yale	-3.2

[a]See General Methods for details.

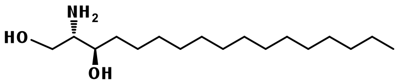

(2*S*,3*R*)-2-amino-1,3-heptadecanediol
$[\alpha]^{25}_{D}$ +1.5 (*c* = 1.0, pyr)

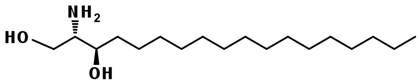

(2*S*,3*R*)-2-amino-1,3-octadecanediol
$[\alpha]^{25}_{D}$ +1.83 (*c* = 1.0, pyr)

S2. 2. High Resolution LC-MS.

Table S3. Summary of LCMS data, analyzed by XCMS[7]

compound	observed mass [M-H]	retention time (min)	fold change[a] (ΔBF2461[e])
**ceramide phosphorylethanolamine (CPE)**	649.487	16.92	ND
	663.503	18.67	3474
	677.520	19.54	3801
	691.534	20.36	4390
	705.549	21.09	1338
**α-GalCer$_{BF}$**	702.547	26.04	259
	716.562	26.66	898
	730.578	27.50	511
	744.593	28.13	604
**ceramide**	526.480	25.70	367
	540.495	26.67	334
	554.514	27.27	46
	568.529	28.08	72
	582.545	28.71	55
	596.555	29.36	10
**phosphatidylethanolamine**[b]	620.424	17.42	16
	634.441	18.00	8
	648.455	19.66	3
	662.471	20.33	7
	676.487	21.45	4
	690.502	22.13	1

[a]Determined by XCMS. [b]phosphatidylethanolamine was analyzed as a control. [e]Sphingolipid-deficient mutant. [f]Strain carries knockout of a gene in an unrelated pathway - used as a control.

7. C.A. Smith, E.J. Want, G.C. Tong, R. Abagyan, and G. Siuzdak. *Analytical Chemistry*, **2006**, *78*, 779–787.

S2.3. Figure S1

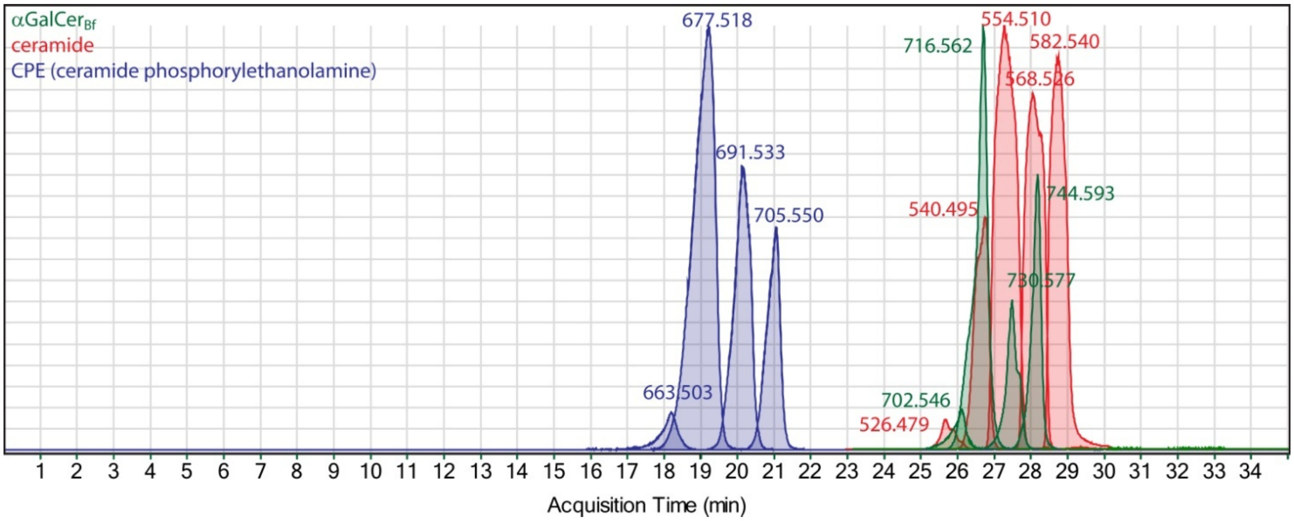

S3. IN VITRO TITRATION

S3.1. Figure S2

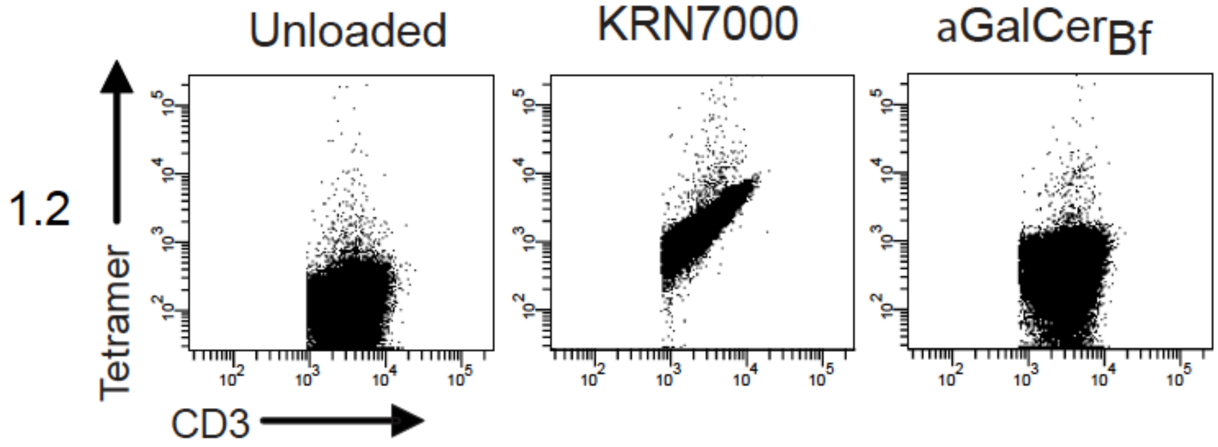

S3.2. Figure S3

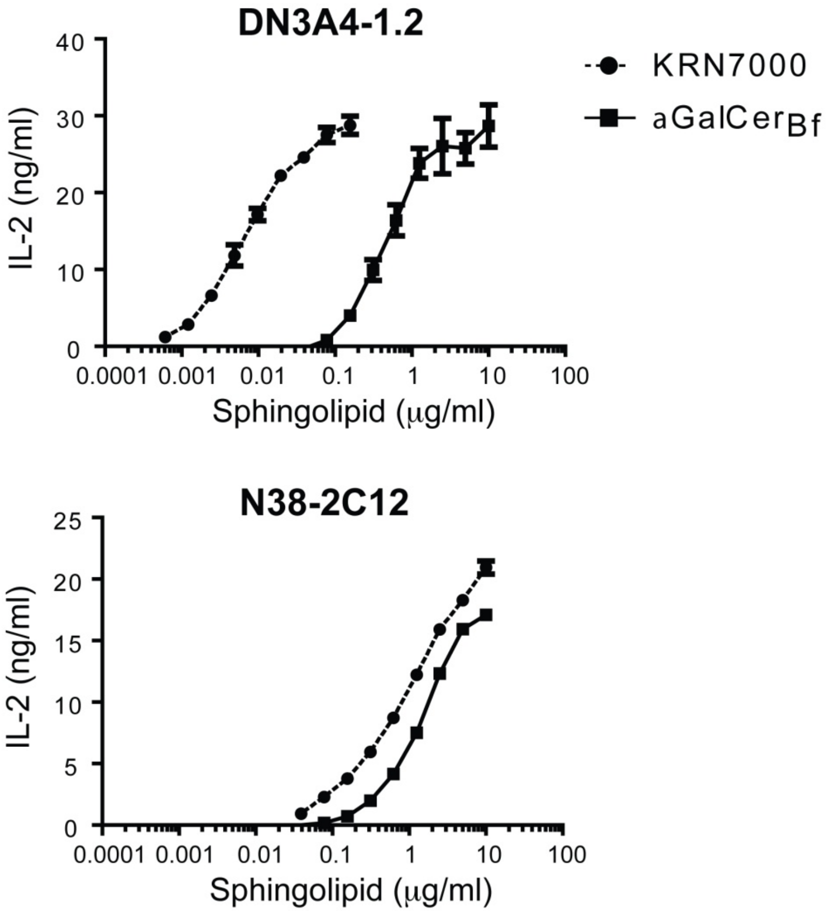

S4. SPECTRAL DATA

S4.1. NMR Data and Assignments for ceramide.

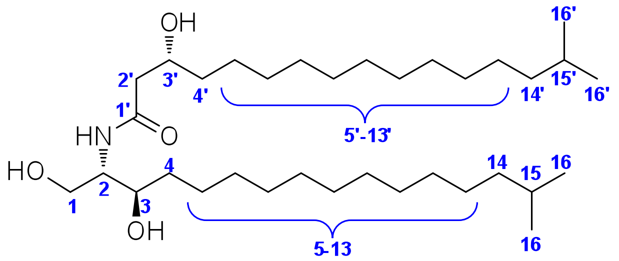

position	δ ^{1}H (multiplicity, J, #H)	δ ^{13}C
NH	7.37 (d, *J* = 8.6 Hz, 1H)	
1	3.74 (dd, *J* = 11.5, 5.3 Hz, 1H)<br>3.66 (dd, J = 11.5, 3.7 Hz, 1H)	61.2
2	3.81 – 3.76 (m, 1H)	54.9
3	3.61 – 3.56 (m, 1H)	72.2
4	1.48 – 1.42 (m, 2H)	34.1
5-13	1.31 – 1.19 (m, 18 H)	31.3-26.0
14	1.14-1.08 (m, 2H)	39.1
15	1.51-1.42 (m, 1H)	28.0
16	0.82 (d, *J* = 6.6 Hz, 6H)	22.4
1'		173.0
2'	2.35 (dd, *J* = 14.5, 3.4 Hz, 1H)<br>2.25 (dd, J = 14.6, 8.9 Hz, 1H)	43.5
3'	3.95 – 3.89 (m, 1H)	68.7
4'	1.47 – 1.40 (m, 2H)	37.3
5'-13'	1.31 – 1.19 (m, 18 H)	31.3-26.0
14'	1.14 – 1.08 (m, 2H)	39.1
15'	1.51-1.42 (m, 1H)	28.0
16’	0.82 (d, *J* = 6.6 Hz, 6H)	22.4

Cosy

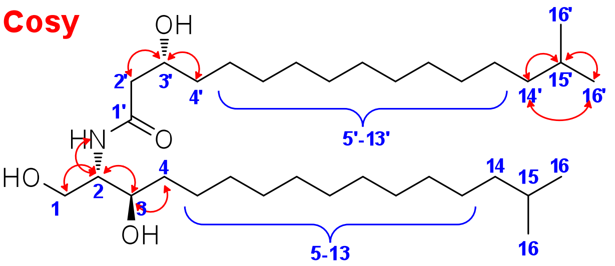

Tocsy

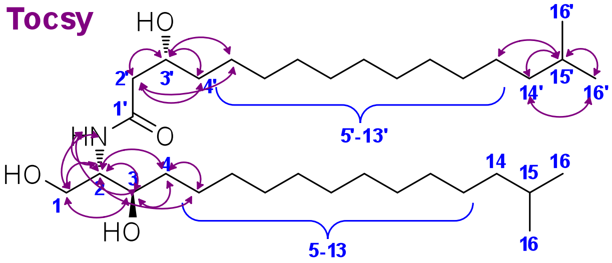

Hmbc

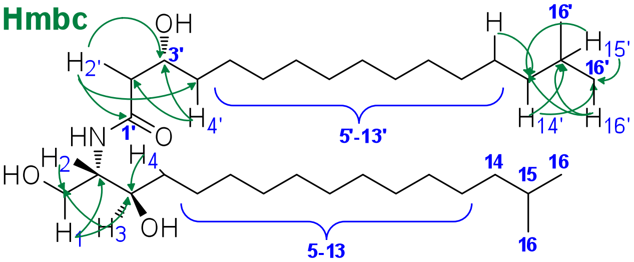

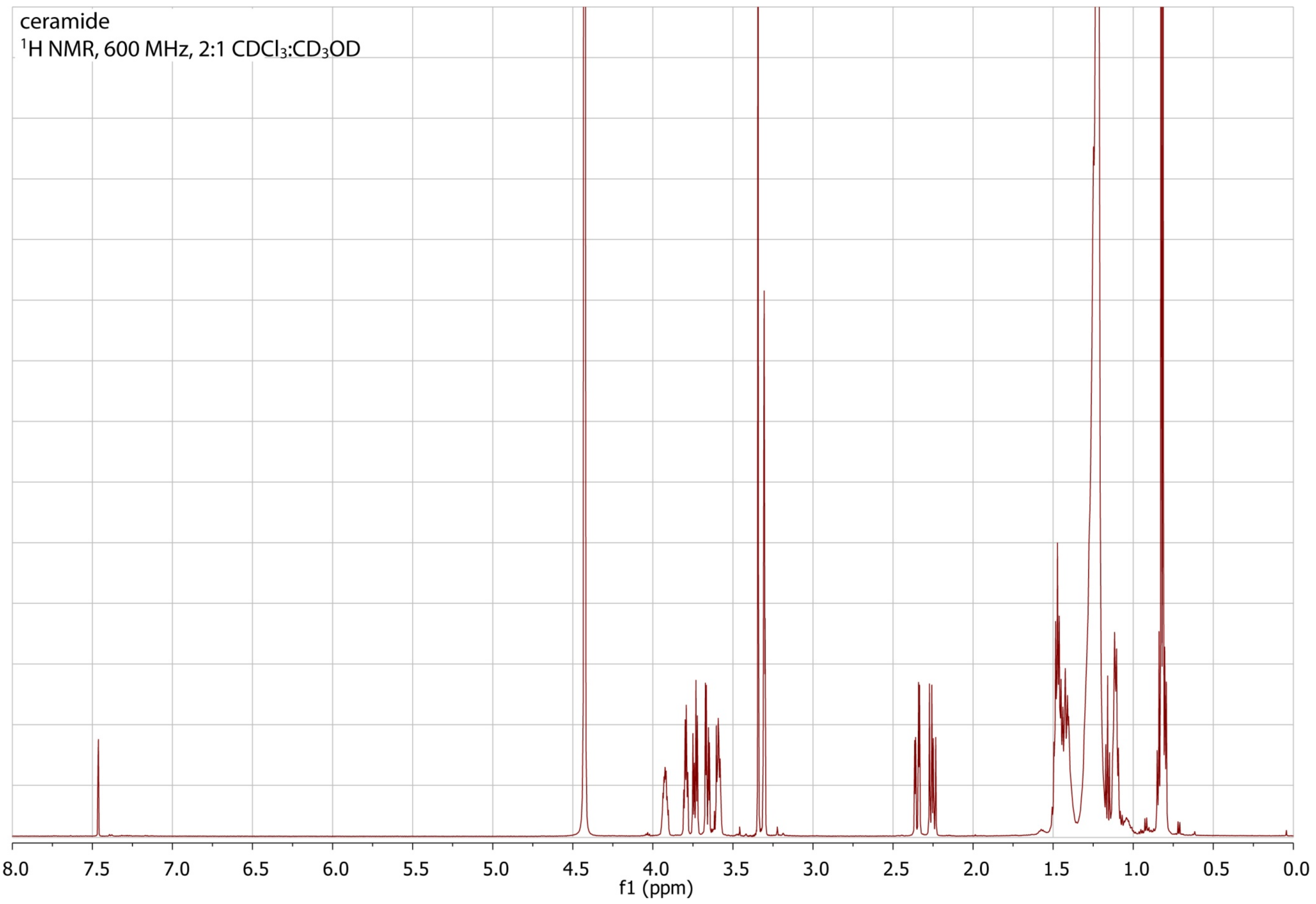
ceramide
1H NMR, 600 MHz, 2:1 CDCl3:CD3OD
8.0 7.5 7.0 6.5 6.0 5.5 5.0 4.5 4.0 3.5 3.0 2.5 2.0 1.5 1.0 0.5 0.0
f1 (ppm)

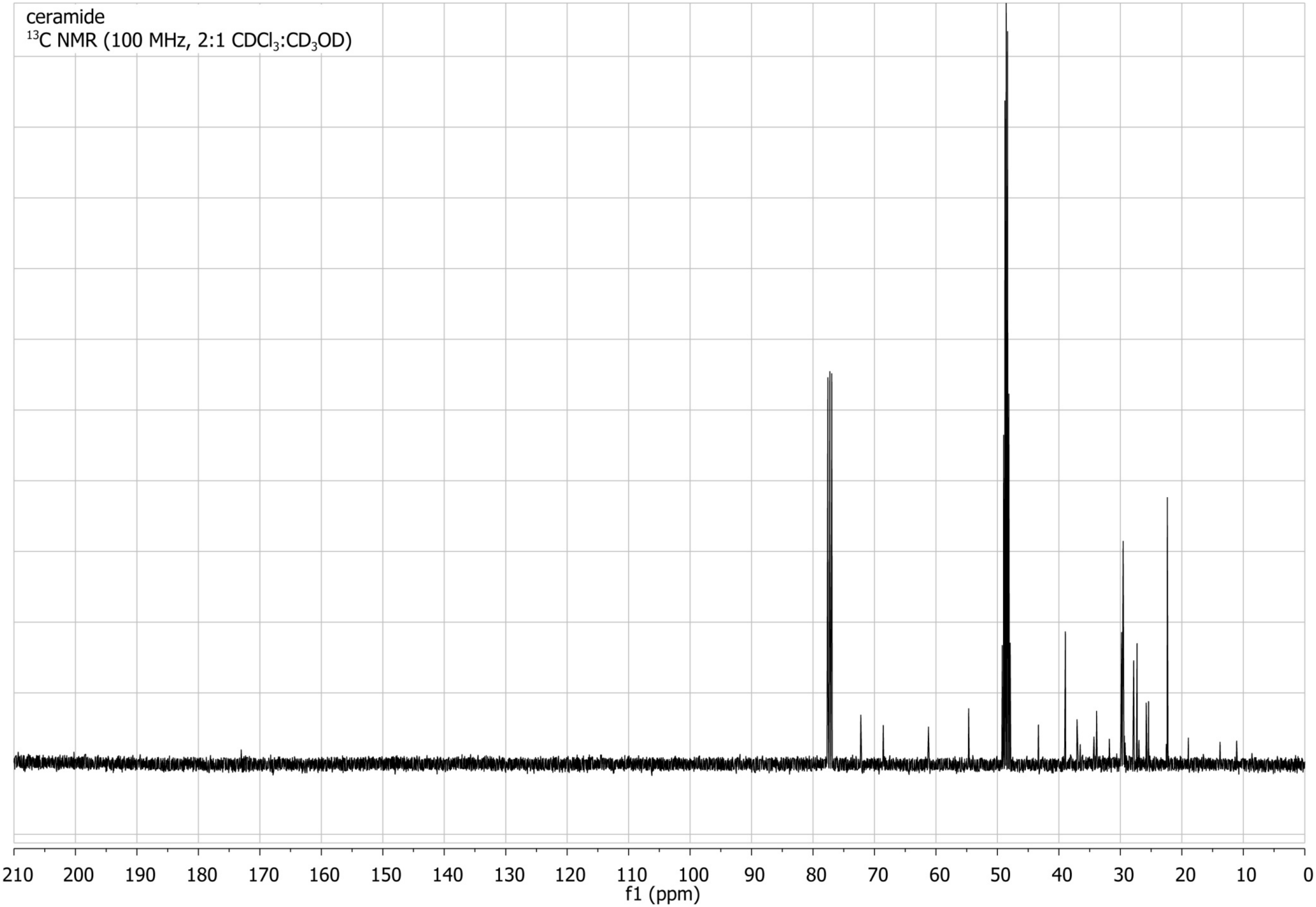
ceramide
^{13}C NMR (100 MHz, 2:1 $CDCl_3$:CD_3OD)
210 200 190 180 170 160 150 140 130 120 110 100 90 80 70 60 50 40 30 20 10 0
f1 (ppm)

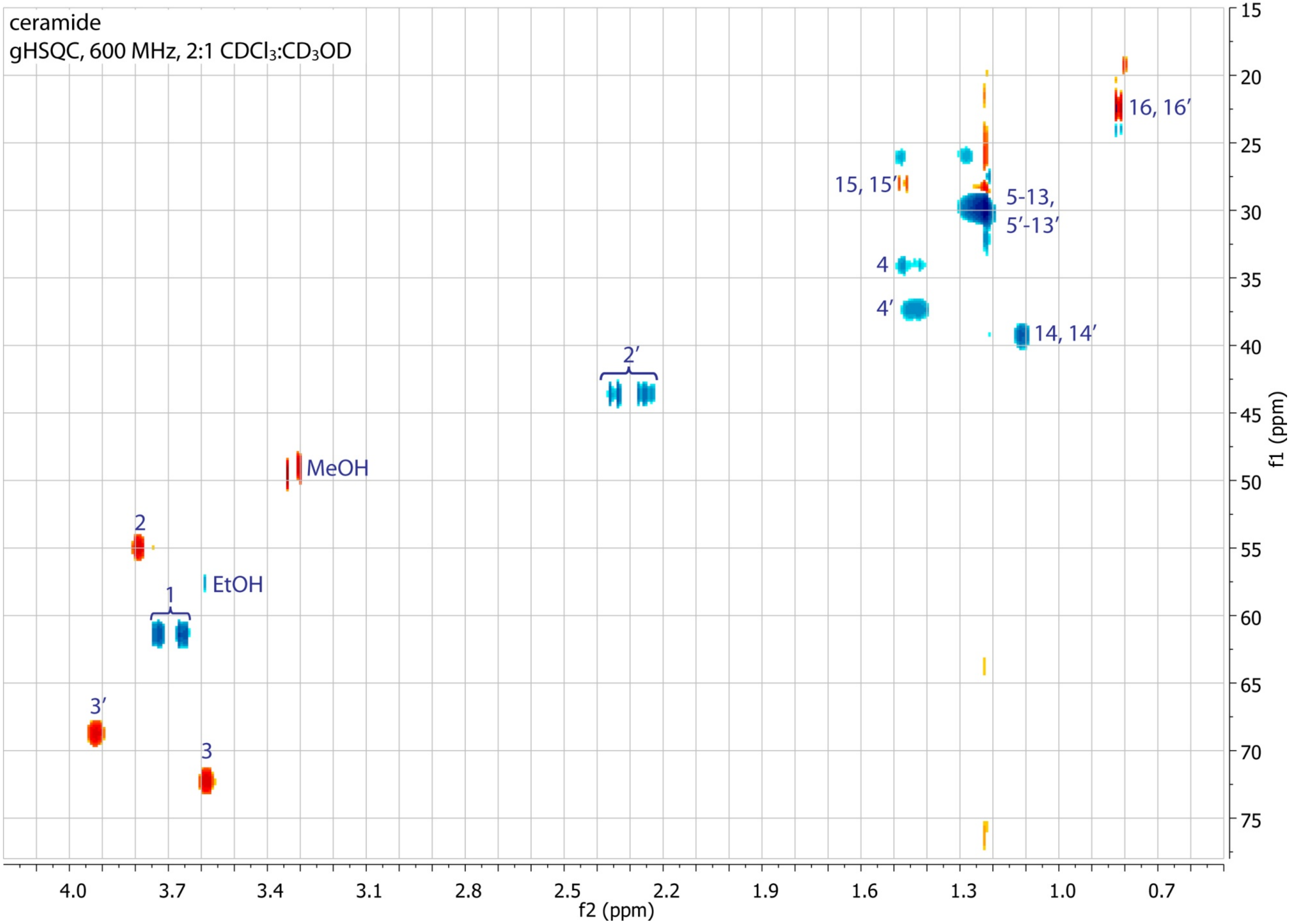
ceramide
gHSQC, 600 MHz, 2:1 $CDCl_3$:CD_3OD
16, 16′
15, 15′
5-13,
5′-13′
4
4′
14, 14′
2′
MeOH
2
EtOH
1
3′
3
f1 (ppm)
f2 (ppm)

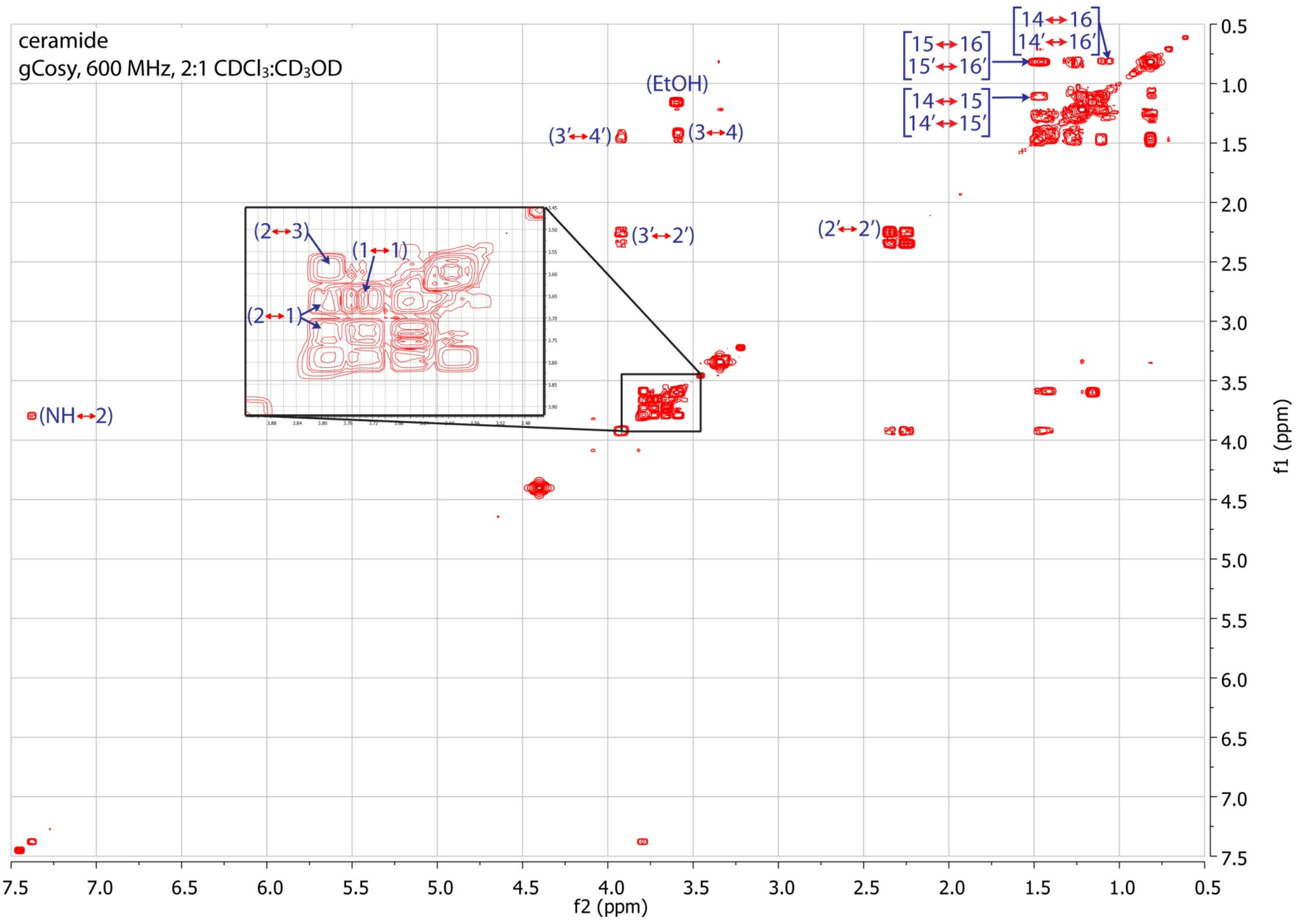
ceramide
gCosy, 600 MHz, 2:1 $CDCl_3$:CD_3OD
(EtOH)
(3′↔4′)
(3↔4)
(3′↔2′)
(2′↔2′)
(2↔3)
(1↔1)
(2↔1)
(NH↔2)
[15↔16 15′↔16′]
[14↔16 14′↔16′]
[14↔15 14′↔15′]
f2 (ppm)
f1 (ppm)

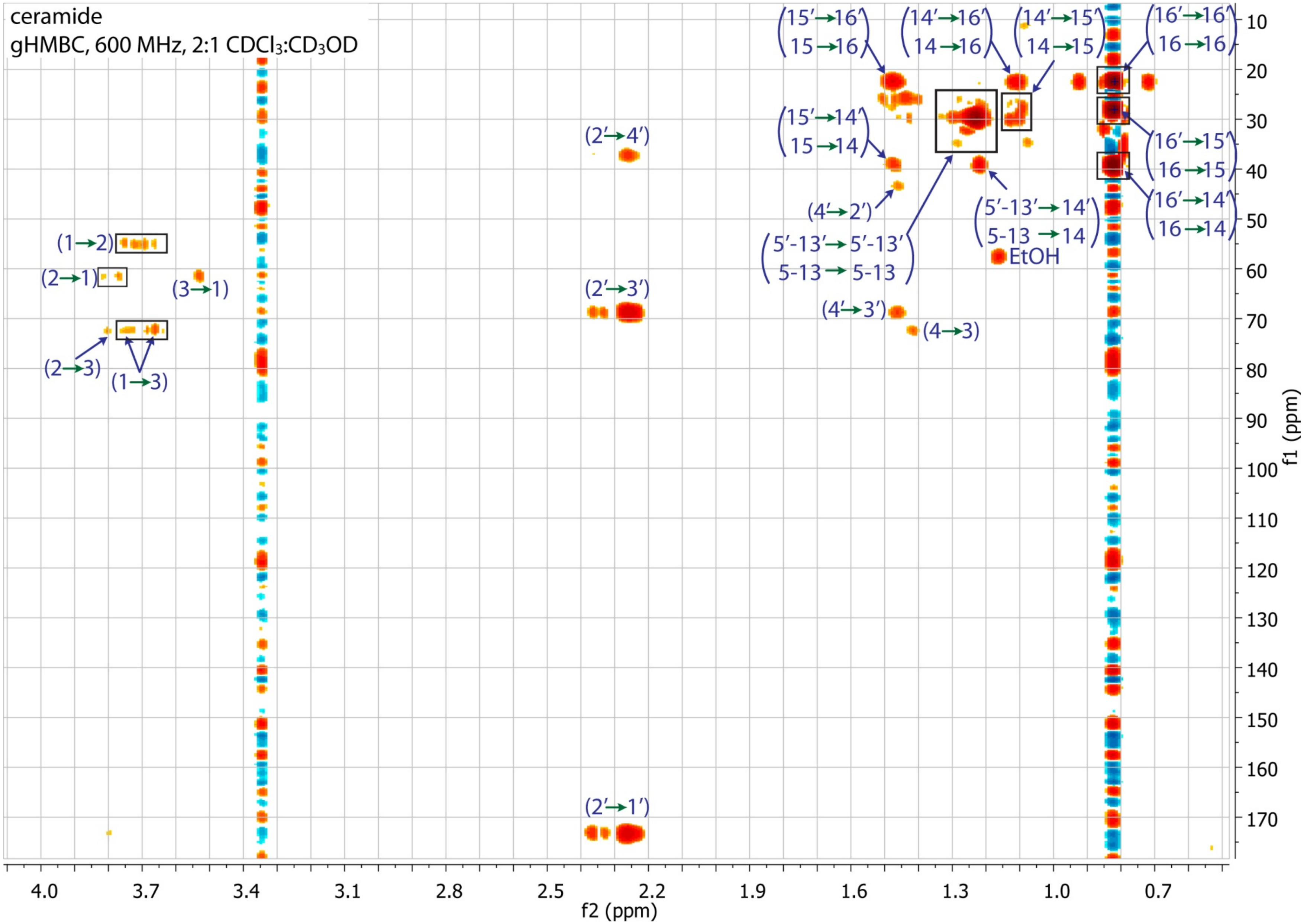
ceramide
gHMBC, 600 MHz, 2:1 CDCl3:CD3OD
(15′→16′) (15→16)
(14′→16′) (14→16)
(14′→15′) (14→15)
(16′→16′) (16→16)
(2′→4′)
(15′→14′) (15→14)
(16′→15′) (16→15)
(4′→2′)
(5′-13′→14′) (5-13 →14)
(16′→14′) (16→14)
(1→2)
(2→1)
(3→1)
(5′-13′→5′-13′) (5-13→5-13)
EtOH
(2′→3′)
(4′→3′)
(4→3)
(2→3)
(1→3)
(2′→1′)
f1 (ppm)
f2 (ppm)

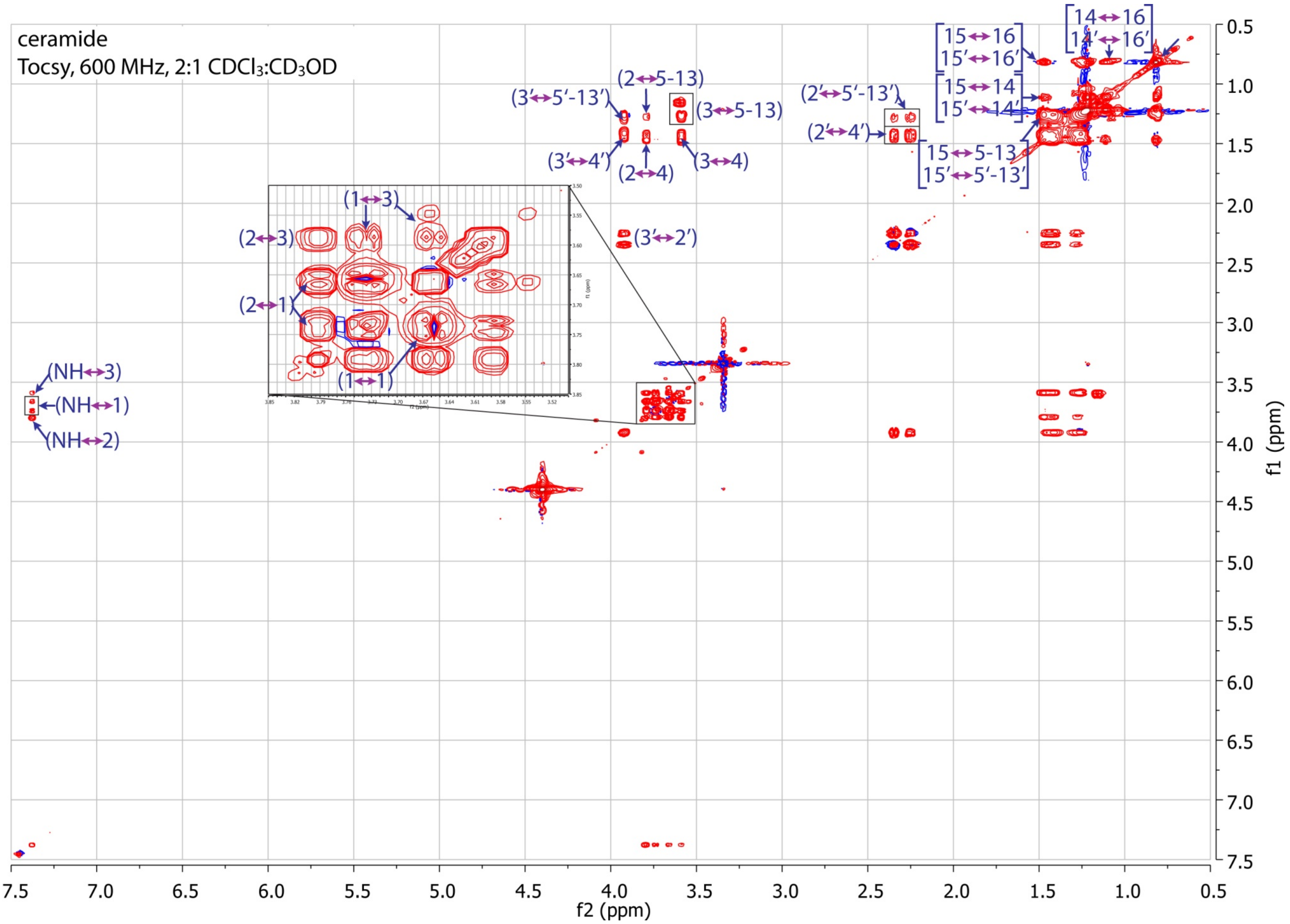
ceramide
Tocsy, 600 MHz, 2:1 CDCl3:CD3OD
(2↔5-13)
(3′↔5′-13′)
(3↔5-13)
(3′↔4′)
(2↔4)
(3↔4)
(2′↔5′-13′)
(2′↔4′)
15↔16
15′↔16′
14↔16
14′↔16′
15↔14
15′↔14′
15↔5-13
15′↔5′-13′
(1↔3)
(2↔3)
(2↔1)
(1↔1)
(3′↔2′)
(NH↔3)
(NH↔1)
(NH↔2)
f2 (ppm)
f1 (ppm)

S4.2. NMR Data and Assignments for α-GalCer$_{Bf}$.

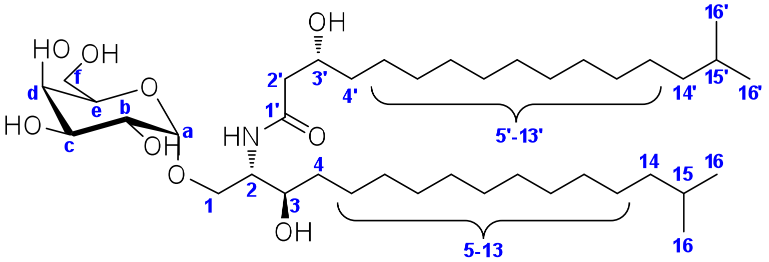

position	δ ^{1}H (multiplicity, J, #H)	δ ^{13}C
**a**	4.64 (d, *J* = 3.3 Hz, 1H)	100.0
**b**	3.50 – 3.46 (m, 1H)	69.1
**b-OH**	4.15 (d, *J* = 7.6 Hz, 1H)	
**c**	3.68 – 3.65 (m, 1H)	69.3
**c-OH**	4.33 (d, *J* = 4.2 Hz, 1H)	
**d**	3.53 – 3.50 (m, 1H)	70.2
**d-OH**	4.47 (d, *J* = 5.4 Hz, 1H)	
**e**	3.59 – 3.55 (m, 1H)	71.6
**f**	3.48 – 3.45 (m, 1H)	61.0
**f**	3.42 – 3.37 (m, 1H)	61.0
**f-OH**	4.48 (d, *J* = 5.4 Hz, 1H)	
**NH**	7.60 (d, *J* = 9.1 Hz, 1H)	
**1**	3.56 – 3.51 (m, 2H)	67.4
**2**	3.73 – 3.69 (m, 1H)	53.4
**3**	3.45 – 3.42 (m, 1H)	69.6
**3-OH**	4.52 (d, *J* = 6.4 Hz, 1H)	
**4**	1.43-1.38 (m, 1H)<br>1.20 – 1.18 (m, 1H)	34.2
**5-13**	1.24 – 1.17 (m, 18H)	31.7-25.6
**14**	1.14 – 1.06 (m, 2 H)	38.9
**15**	1.52-1.42 (m, 1H)	27.9
**16**	0.82 (d, *J* = 6.6 Hz, 6H)	22.9
**1’**		170.8
**2'**	2.17 (ddd, *J* = 26.3, 13.8, 6.6 Hz, 2H)	44.5
**3'**	3.79 – 3.73 (m, 1H)	67.9
**3'-OH**	4.58 (d, *J* = 4.9 Hz, 1H)	
**4'**	1.33 – 1.31 (m, 1H)<br>1.27 – 1.25 (m, 1H)	37.0
**5'-13'**	1.24 – 1.17 (m, 18H)	31.7-25.6
**14'**	1.14 – 1.06 (m, 2 H)	38.9
**15'**	1.52-1.42 (m, 1H)	27.9
**16'**	0.82 (d, *J* = 6.6 Hz, 6H)	22.9

Cosy

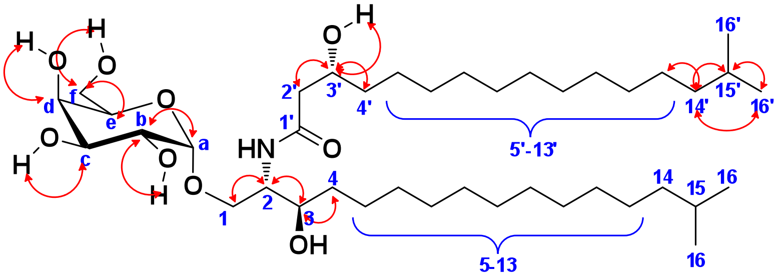

Tocsy

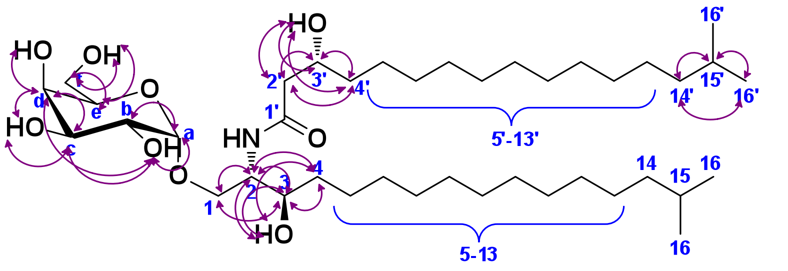

Hmbc

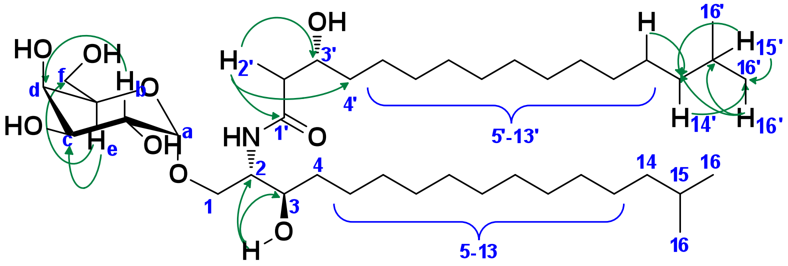

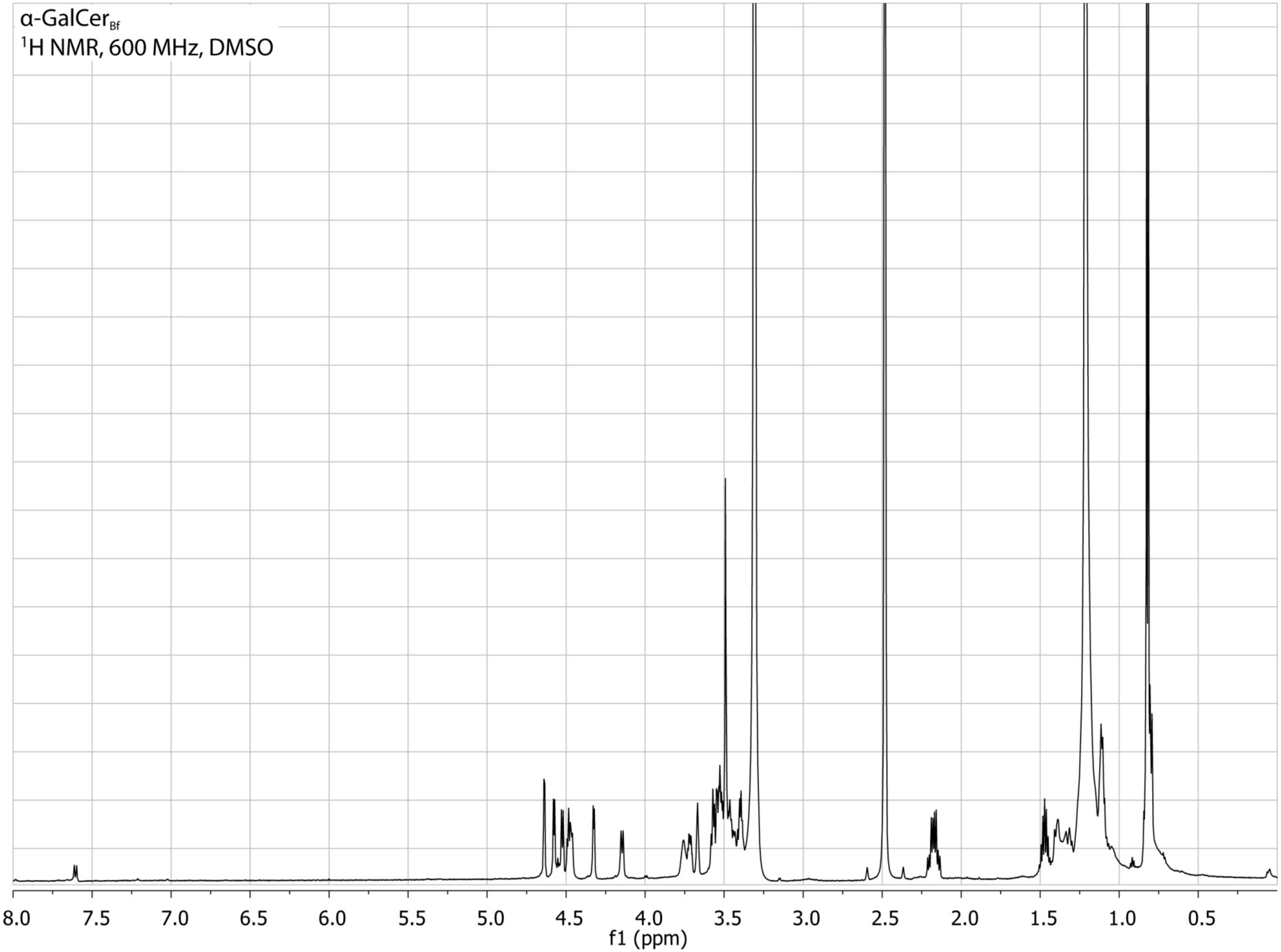

α-GalCer$_{Bf}$
^{1}H NMR, 600 MHz, DMSO
8.0
7.5
7.0
6.5
6.0
5.5
5.0
4.5
4.0
3.5
3.0
2.5
2.0
1.5
1.0
0.5
f1 (ppm)

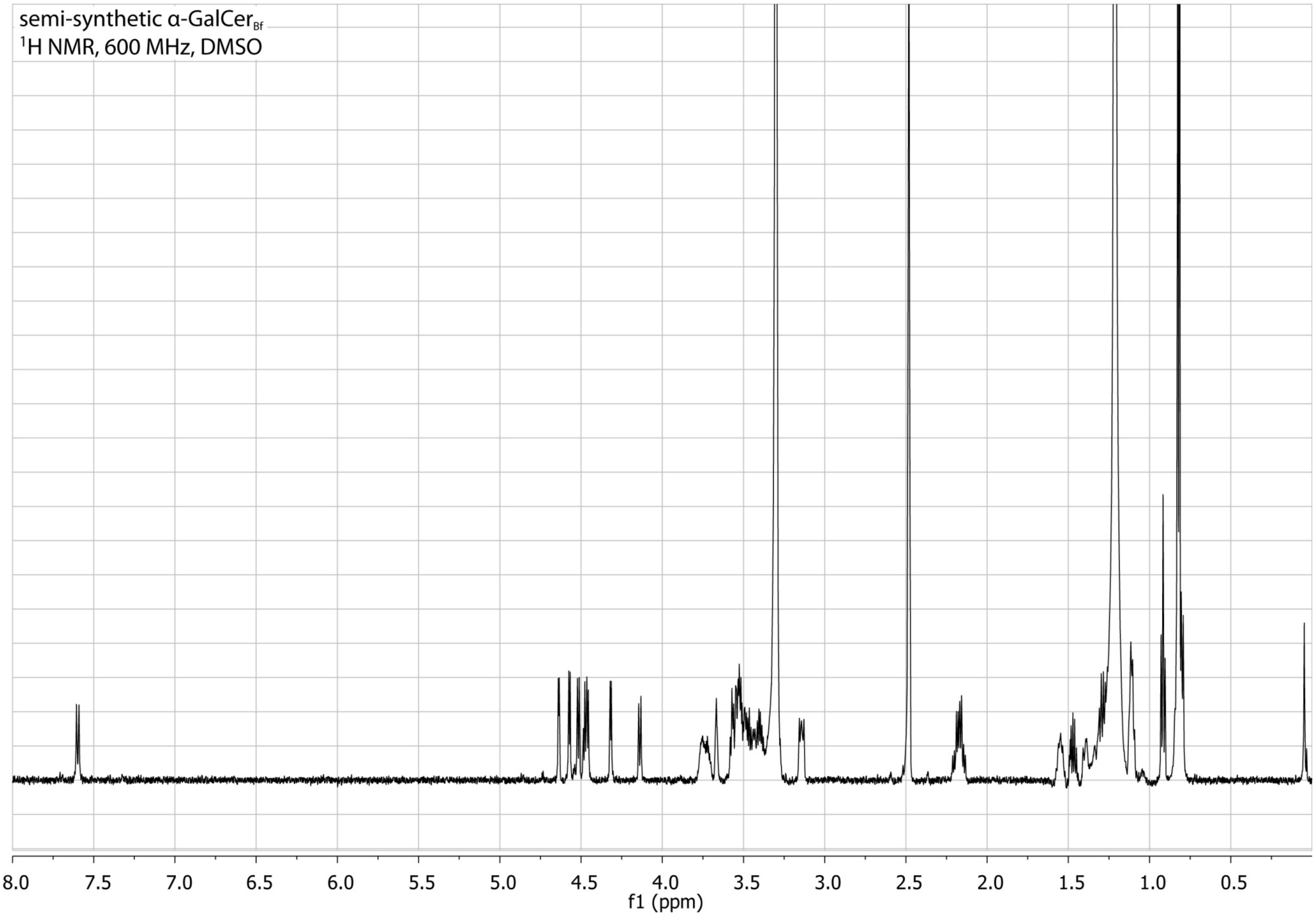
semi-synthetic α-GalCer$_{Bf}$
^{1}H NMR, 600 MHz, DMSO
8.0 7.5 7.0 6.5 6.0 5.5 5.0 4.5 4.0 3.5 3.0 2.5 2.0 1.5 1.0 0.5
f1 (ppm)

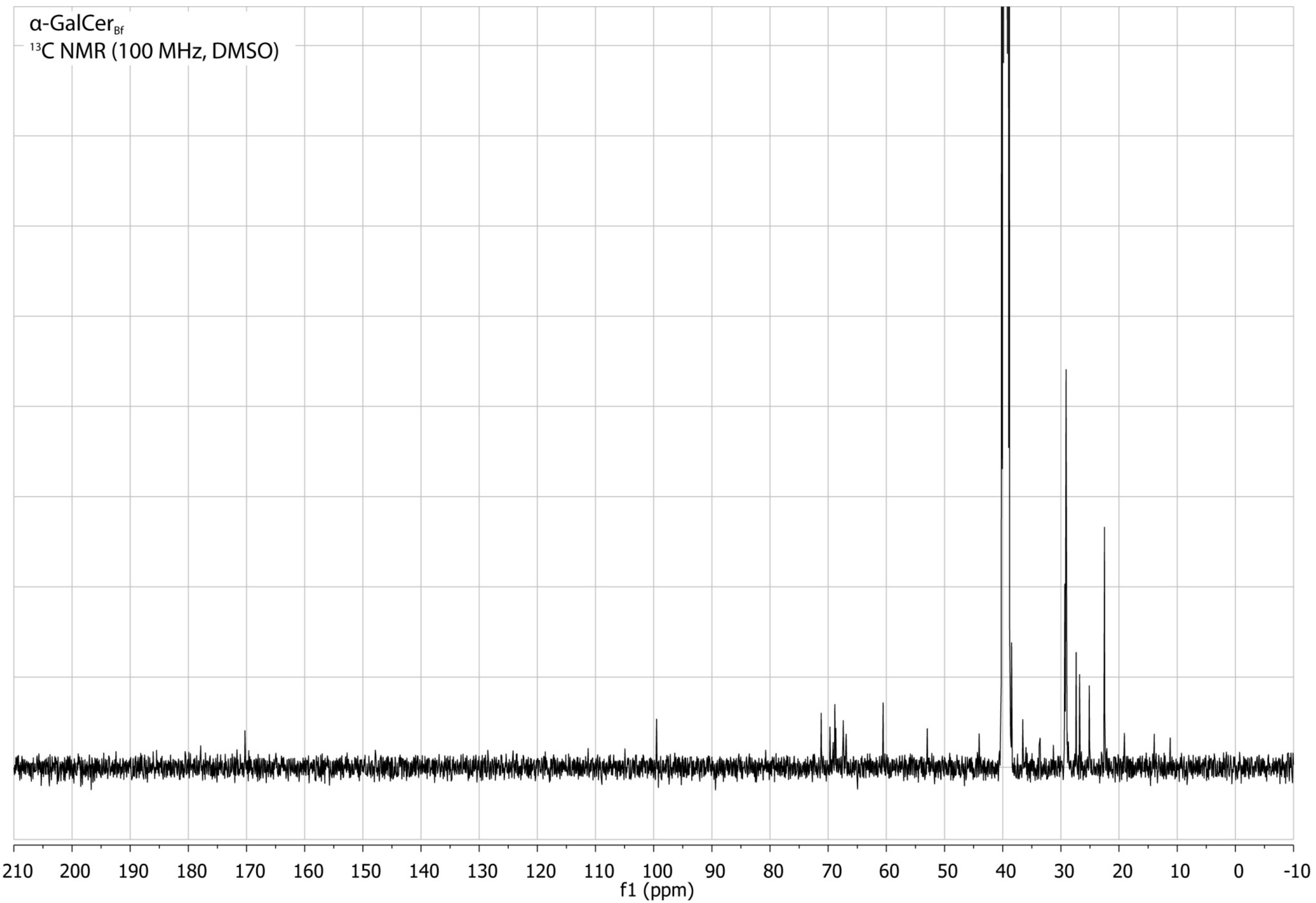

α-GalCer$_{Bf}$
^{13}C NMR (100 MHz, DMSO)
210 200 190 180 170 160 150 140 130 120 110 100 90 80 70 60 50 40 30 20 10 0 -10
f1 (ppm)

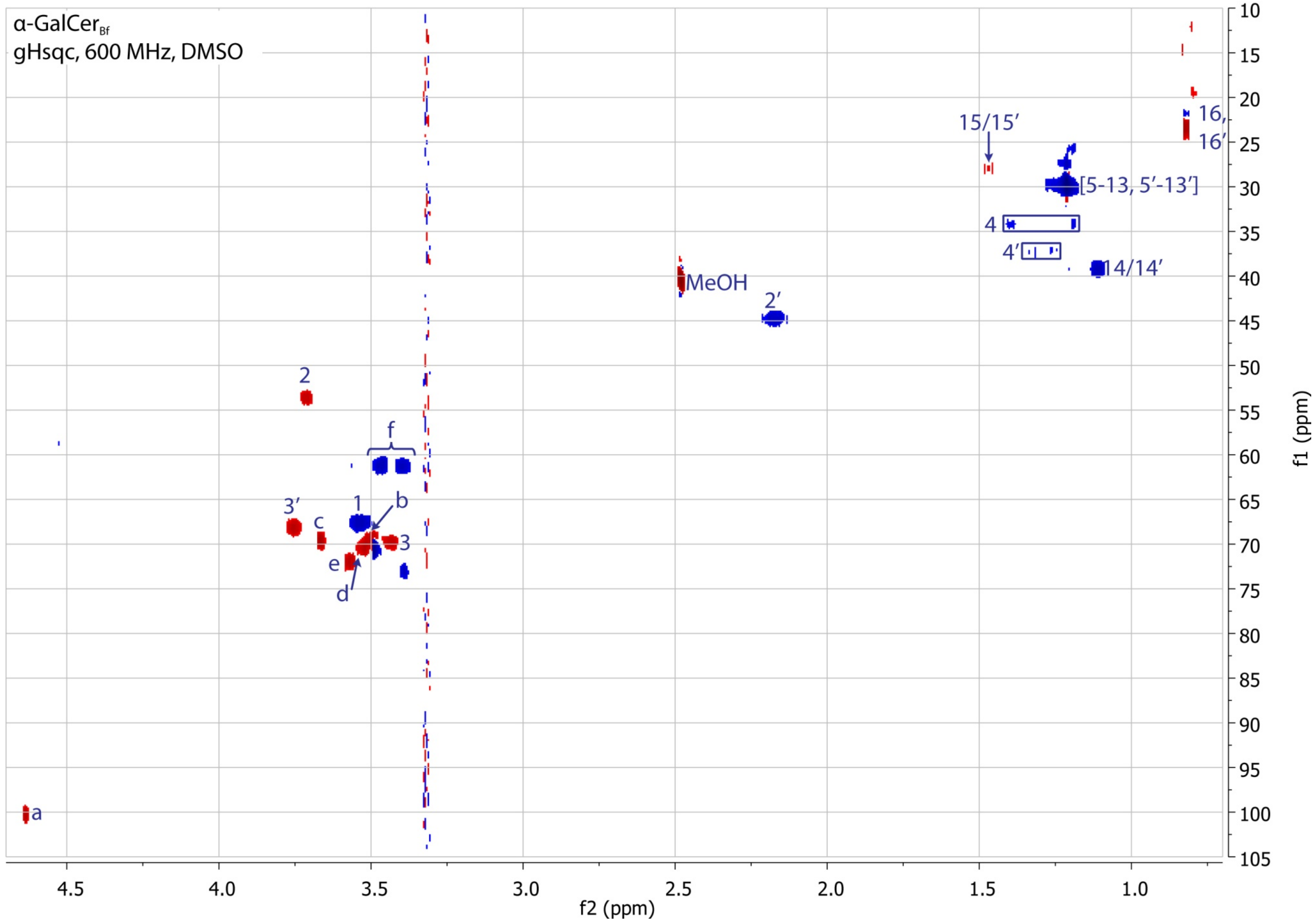
α-GalCer$_{Bf}$
gHsqc, 600 MHz, DMSO
15/15′
16,
16′
[5-13, 5′-13′]
4
4′
14/14′
MeOH
2′
2
f
3′
c
1
b
3
e
d
a
f2 (ppm)
f1 (ppm)

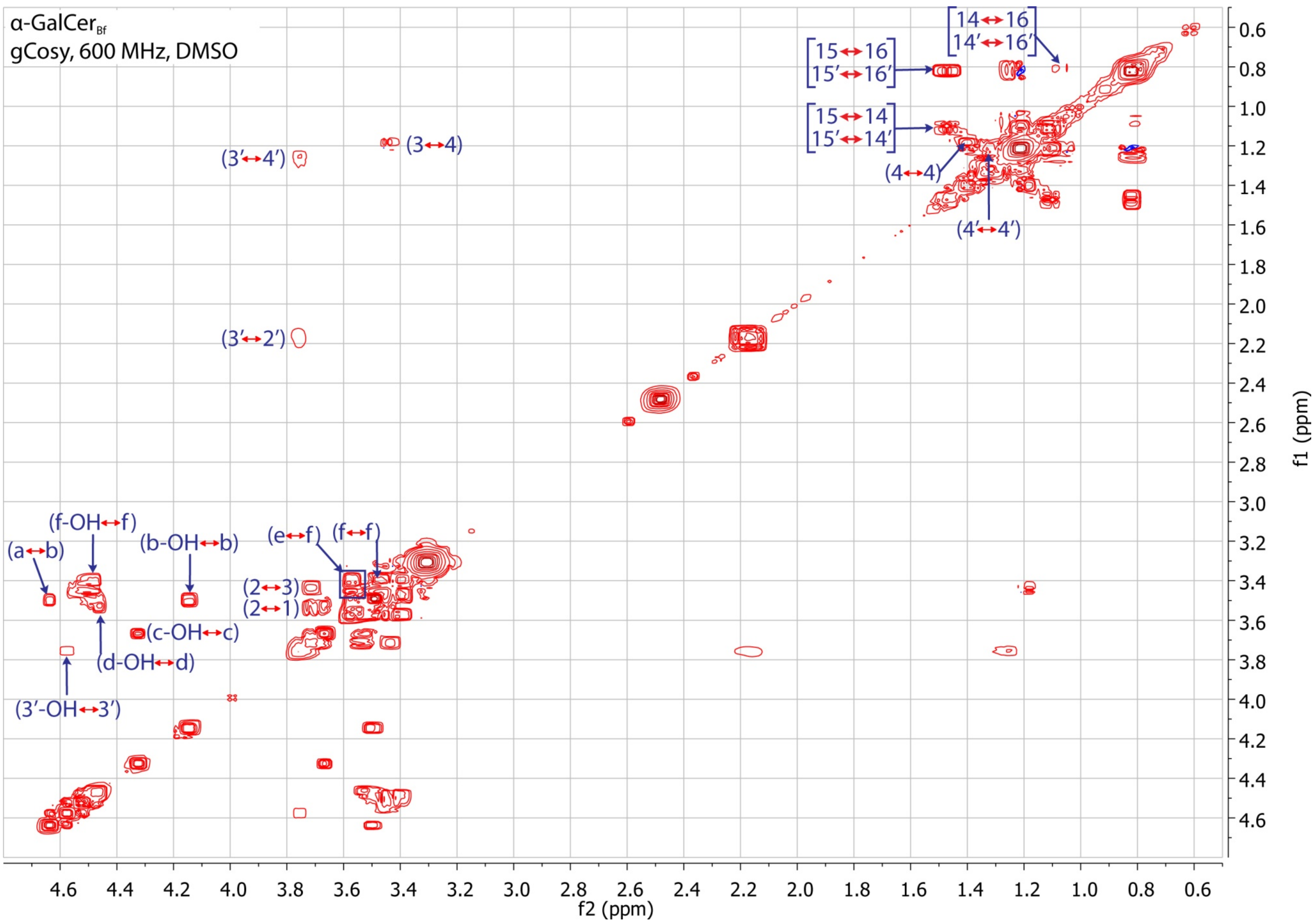
α-GalCer$_{Bf}$
gCosy, 600 MHz, DMSO
14↔16
14′↔16′
15↔16
15′↔16′
15↔14
15′↔14′
(4↔4)
(4′↔4′)
(3↔4)
(3′↔4′)
(3′↔2′)
(a↔b)
(f-OH↔f)
(b-OH↔b)
(e↔f)
(f↔f)
(2↔3)
(2↔1)
(c-OH↔c)
(d-OH↔d)
(3′-OH↔3′)
f2 (ppm)
f1 (ppm)

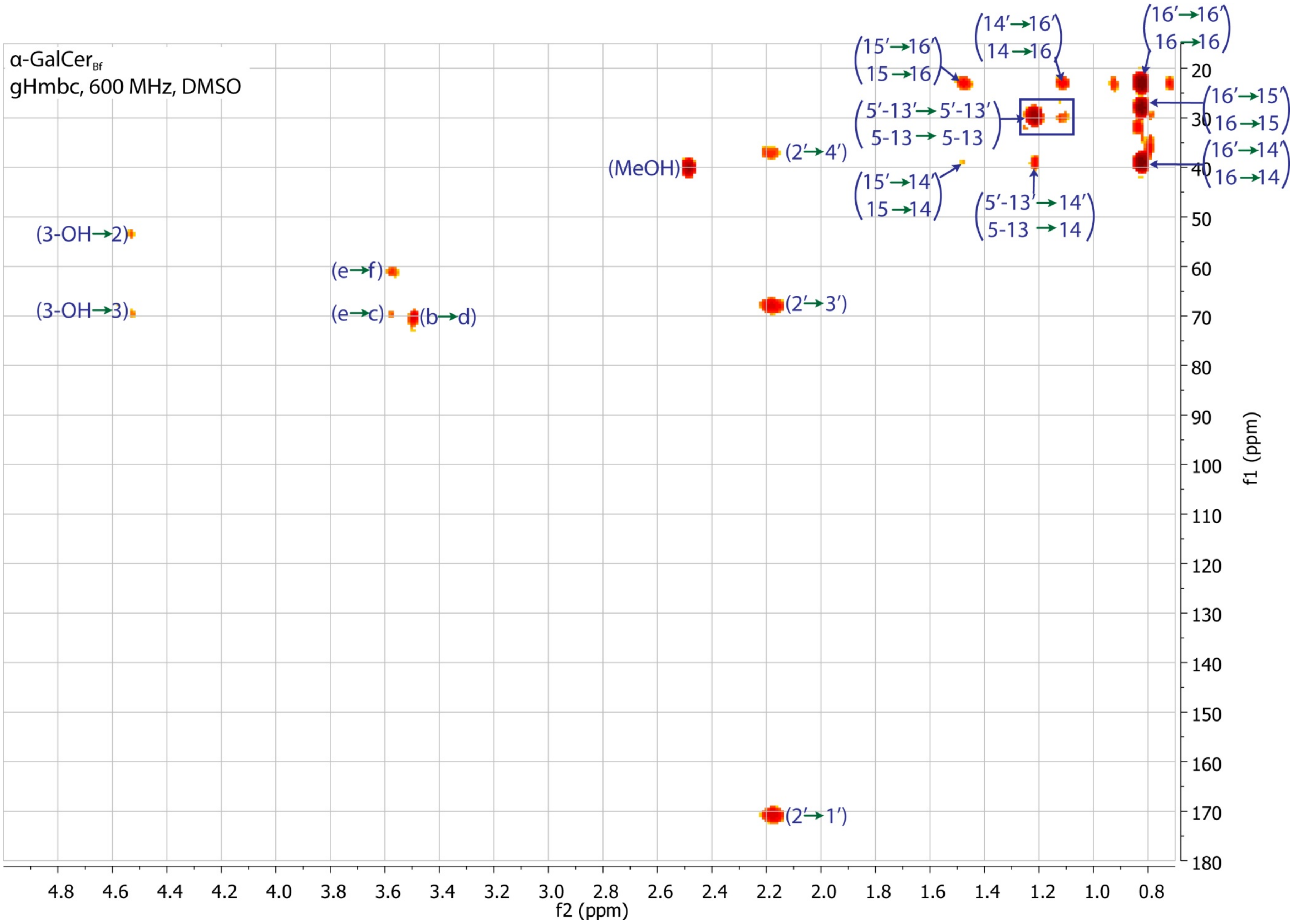

α-GalCer$_{Bf}$
gHmbc, 600 MHz, DMSO
(15′→16′ 15→16)
(14′→16′ 14→16)
(16′→16′ 16→16)
(16′→15′ 16→15)
(5′-13′→5′-13′ 5-13→5-13)
(2′→4′)
(MeOH)
(16′→14′ 16→14)
(15′→14′ 15→14)
(5′-13′→14′ 5-13→14)
(3-OH→2)
(e→f)
(3-OH→3)
(e→c)
(b→d)
(2′→3′)
(2′→1′)
f1 (ppm)
f2 (ppm)

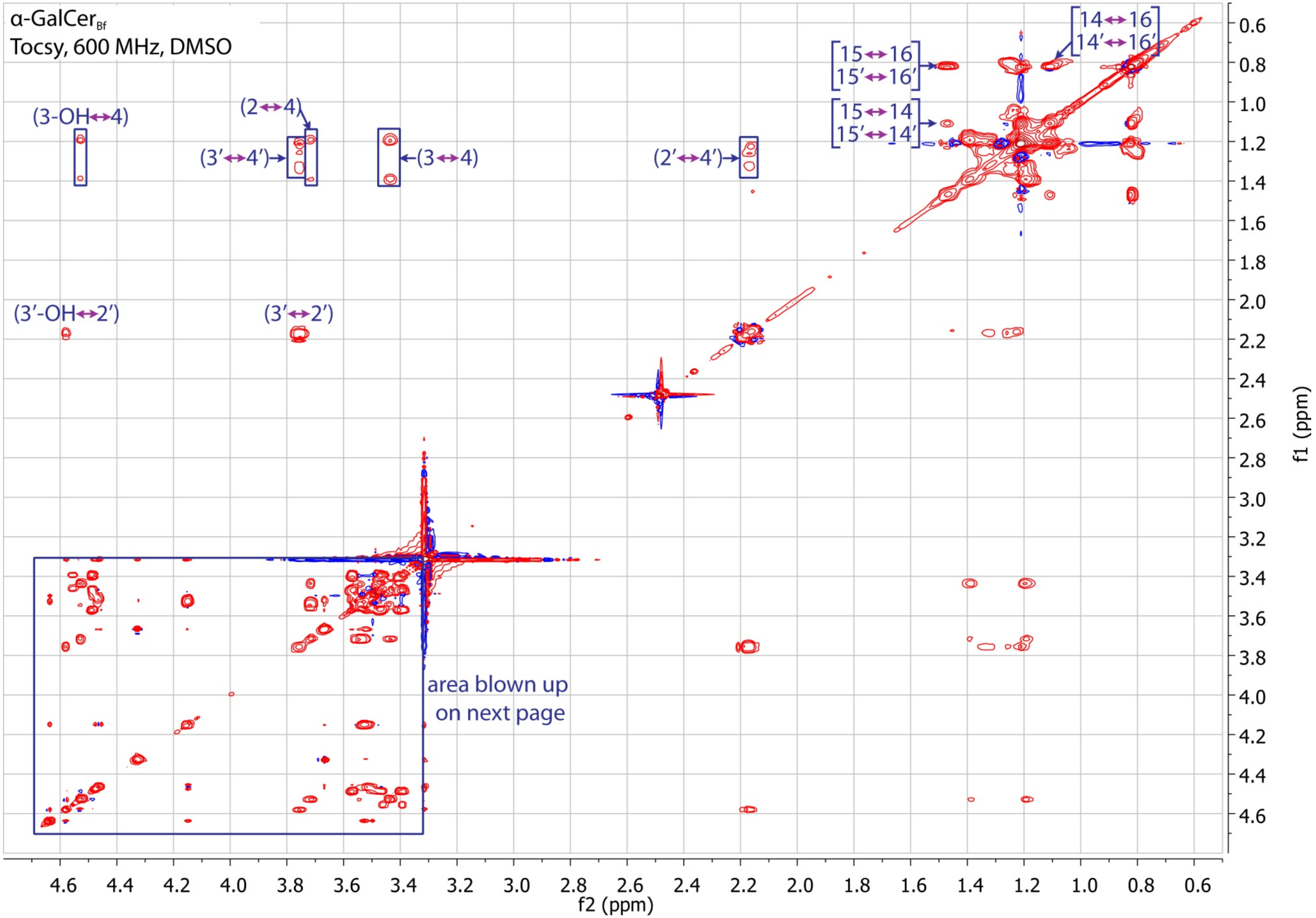
α-GalCer$_{Bf}$
Tocsy, 600 MHz, DMSO
(3-OH↔4)
(2↔4)
(3′↔4′)
(3↔4)
(2′↔4′)
15↔16
15′↔16′
14↔16
14′↔16′
15↔14
15′↔14′
(3′-OH↔2′)
(3′↔2′)
area blown up
on next page
f2 (ppm)
f1 (ppm)

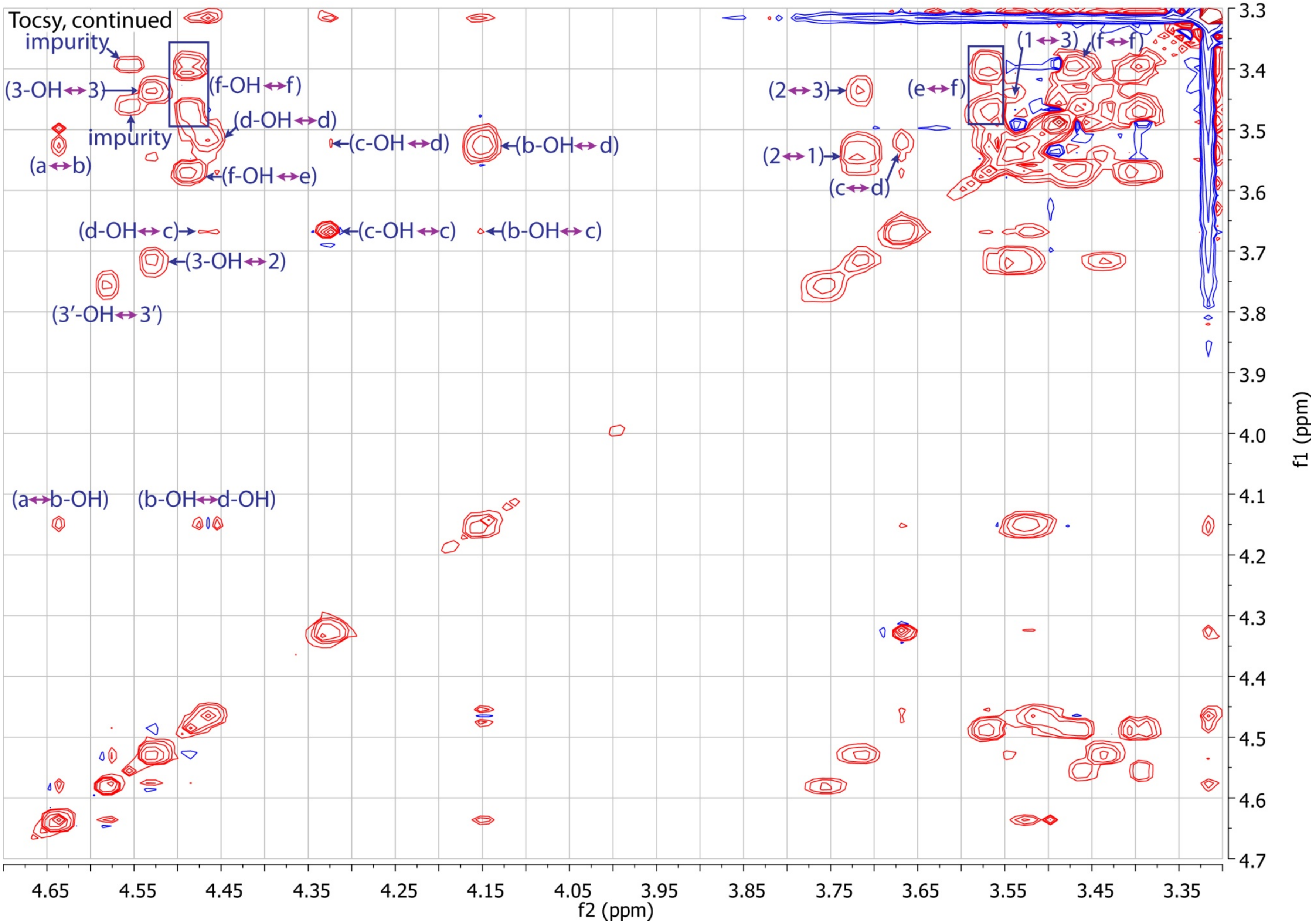
Tocsy, continued
impurity
(3-OH↔3)
impurity
(a↔b)
(f-OH↔f)
(d-OH↔d)
(f-OH↔e)
(c-OH↔d)
(b-OH↔d)
(d-OH↔c)
(c-OH↔c)
(b-OH↔c)
(3-OH↔2)
(3′-OH↔3′)
(2↔3)
(e↔f)
(1↔3)
(f↔f)
(2↔1)
(c↔d)
(a↔b-OH)
(b-OH↔d-OH)
f2 (ppm)
f1 (ppm)

S4.3. NMR Data and Assignments for ceramide phosphorylethanolamine.

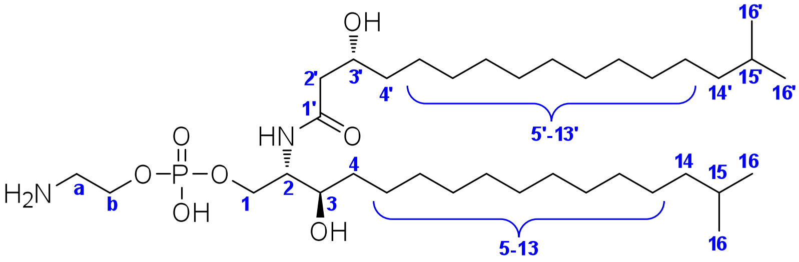

position	δ ^{1}H (multiplicity, J, #H)	δ ^{13}C
**a**	3.09 – 2.99 (m, 2H)	40.1
**b**	4.04 – 3.94 (m, 2H)	61.5
**NH**		
**1**	4.11 – 4.05 (m, 1H)<br>3.94 – 3.88 (m, 1H)	64.3
**2**	3.88 – 3.84 (m, 1H)	53.9
**3**	3.59-3.53 (m, 1H)	69.7
**4**	1.38-1.36 (m, 1H)<br>1.52-1.50 (m, 1H)	33.1
**5-13**	1.33-1.21 (m, 18H)	31.6-25.3
**14**	1.14-1.11 (m, 2H)	38.8
**15**	1.49-1.47 (m, 1H)	27.1
**16**	0.82 (d, J = 6.6 Hz, 6H)	22.0
**1'**		172.6
**2'**	2.34 (dd, J = 14.6, 3.3 Hz, 1H)<br>2.25 (dd, J = 14.6, 9.3 Hz, 1H)	43.2
**3'**	3.93 – 3.89 (m, 1H)	68.3
**4'**	1.44-1.38 (m, 2H)	37.0
**5'-13'**	1.33-1.21 (m, 18H)	31.6-25.3
**14'**	1.14-1.11 (m, 2H)	38.8
**15'**	1.49-1.47 (m, 1H)	27.1
**16'**	0.82 (d, J = 6.6 Hz, 6H)	22.0

Cosy

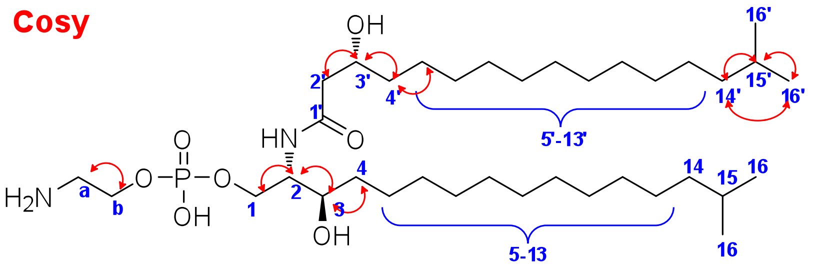

Tocsy

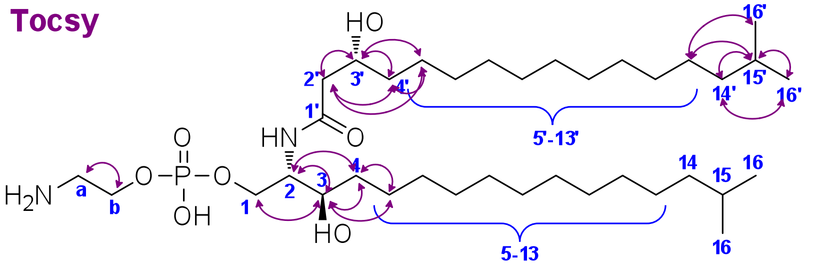

Hmbc

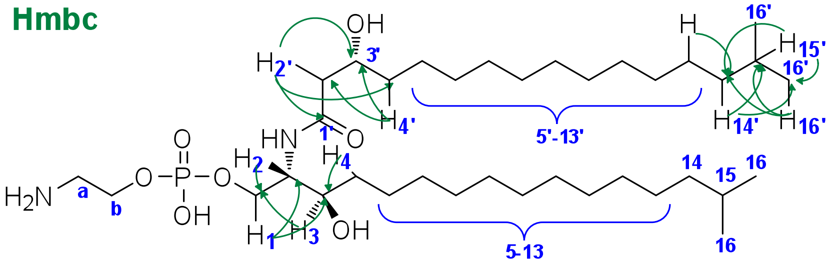

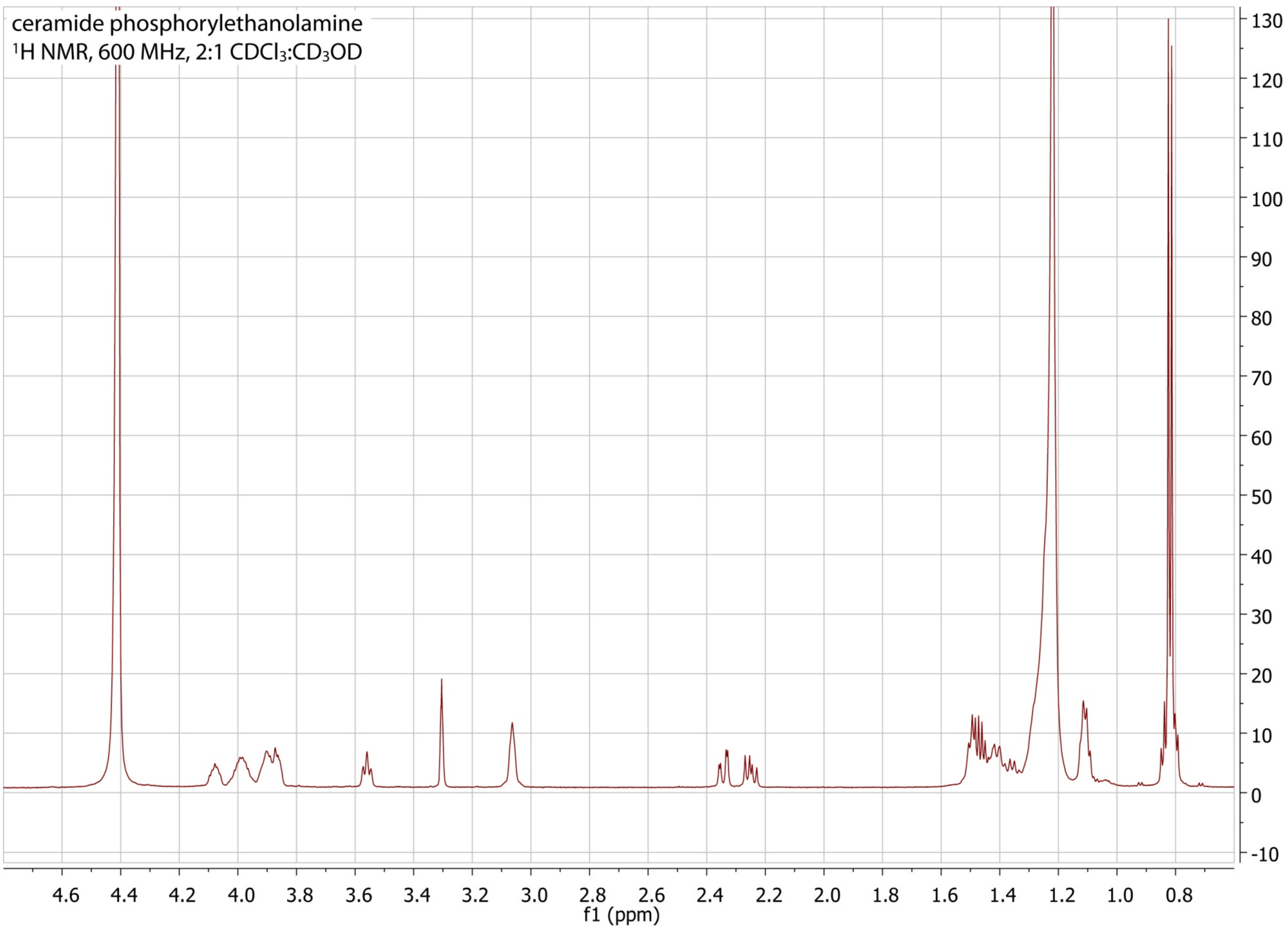

ceramide phosphorylethanolamine
¹H NMR, 600 MHz, 2:1 CDCl₃:CD₃OD
4.6
4.4
4.2
4.0
3.8
3.6
3.4
3.2
3.0
2.8
2.6
2.4
2.2
2.0
1.8
1.6
1.4
1.2
1.0
0.8
f1 (ppm)
130
120
110
100
90
80
70
60
50
40
30
20
10
0
-10

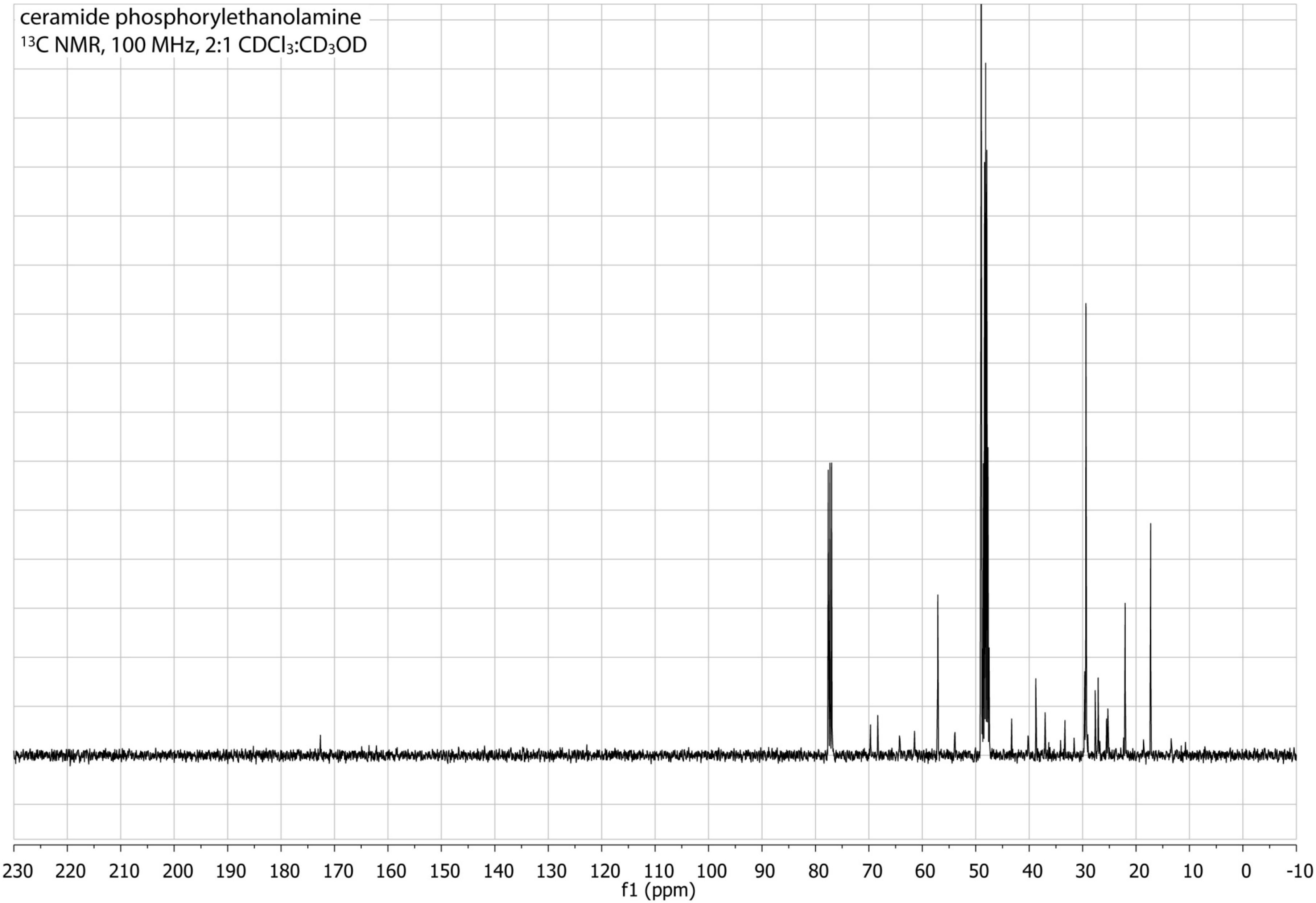
ceramide phosphorylethanolamine
13C NMR, 100 MHz, 2:1 CDCl3:CD3OD
230 220 210 200 190 180 170 160 150 140 130 120 110 100 90 80 70 60 50 40 30 20 10 0 -10
f1 (ppm)

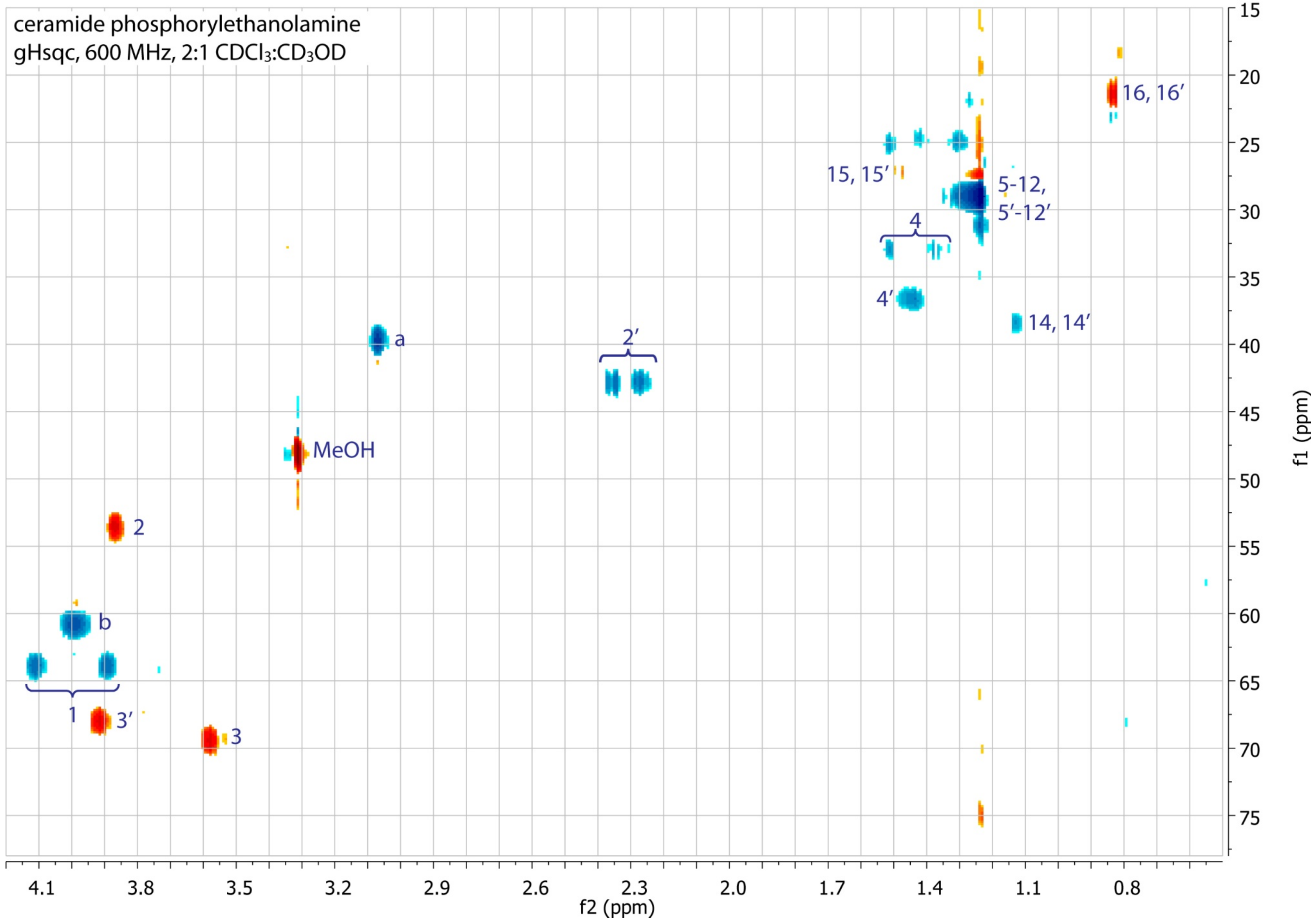

ceramide phosphorylethanolamine
gHsqc, 600 MHz, 2:1 CDCl3:CD3OD
16, 16′
15, 15′
5-12,
5′-12′
4
4′
14, 14′
a
2′
MeOH
2
b
1
3′
3
f2 (ppm)
f1 (ppm)

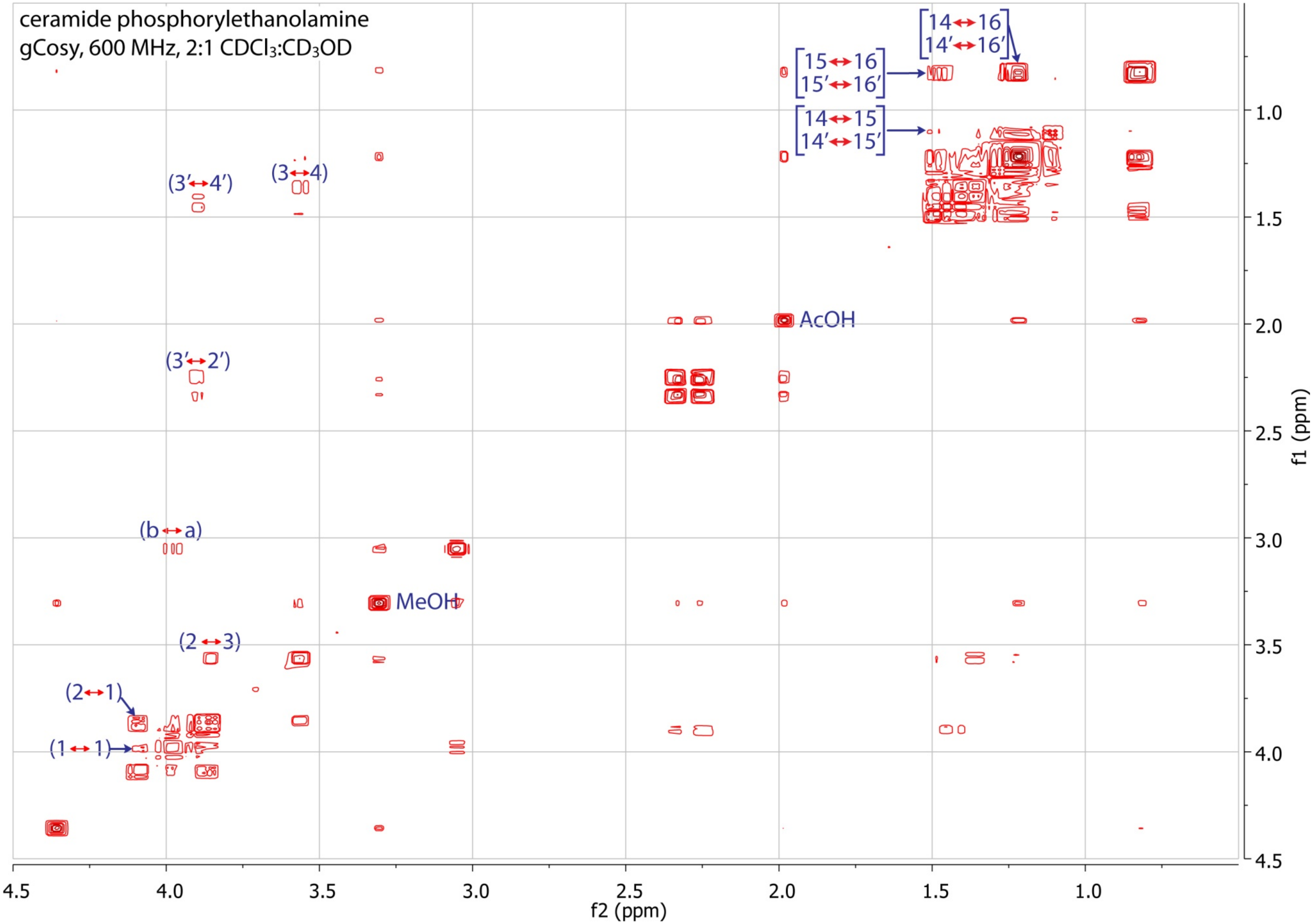

ceramide phosphorylethanolamine
gCosy, 600 MHz, 2:1 CDCl3:CD3OD
14↔16
14′↔16′
15↔16
15′↔16′
14↔15
14′↔15′
(3′↔4′)
(3↔4)
AcOH
(3′↔2′)
(b↔a)
MeOH
(2↔3)
(2↔1)
(1↔1)
f1 (ppm)
f2 (ppm)
4.5
4.0
3.5
3.0
2.5
2.0
1.5
1.0

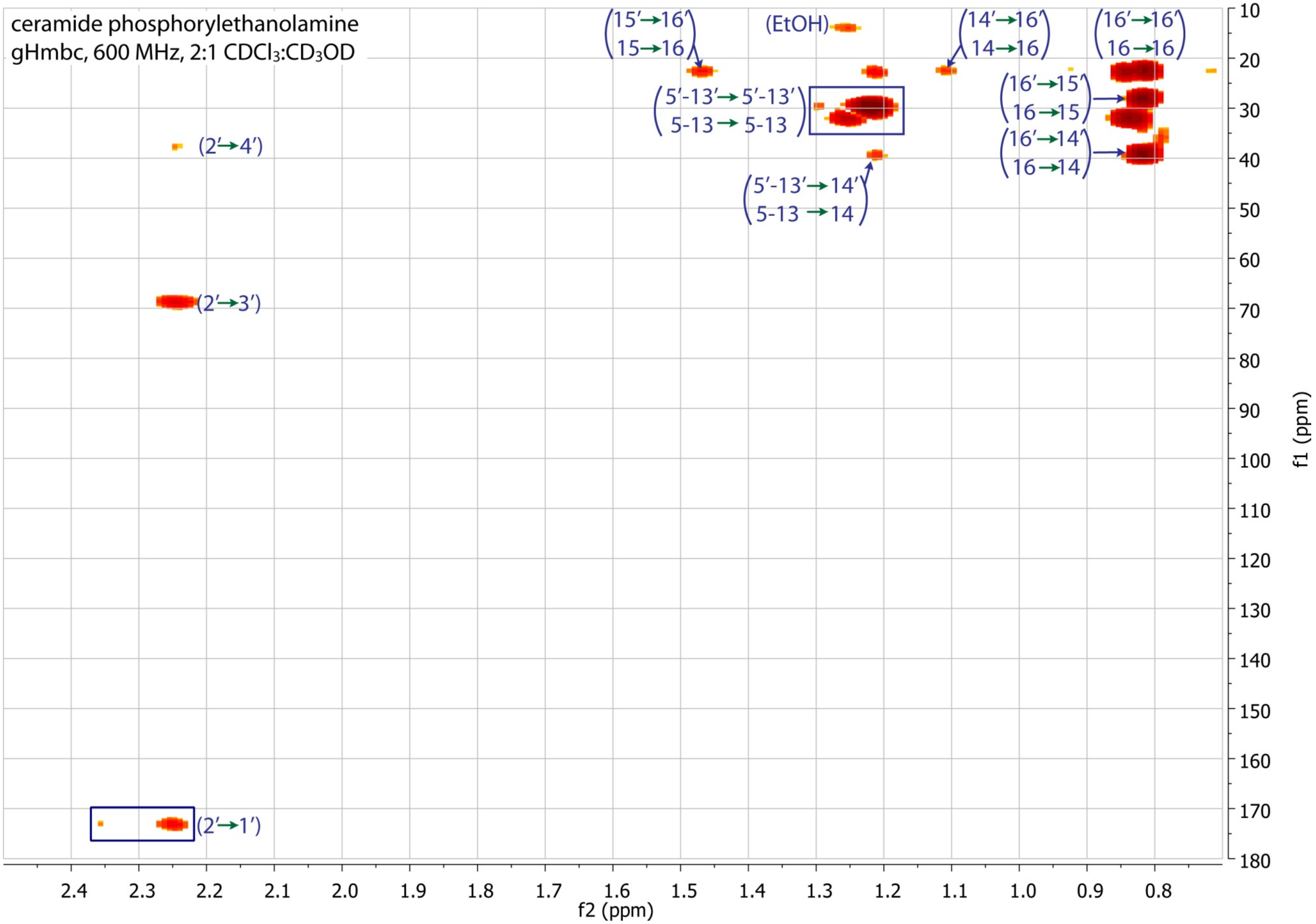
ceramide phosphorylethanolamine
gHmbc, 600 MHz, 2:1 CDCl3:CD3OD
(15′→16′ 15→16)
(EtOH)
(14′→16′ 14→16)
(16′→16′ 16→16)
(5′-13′→5′-13′ 5-13→5-13)
(16′→15′ 16→15)
(16′→14′ 16→14)
(2′→4′)
(5′-13′→14′ 5-13→14)
(2′→3′)
(2′→1′)
f1 (ppm)
f2 (ppm)

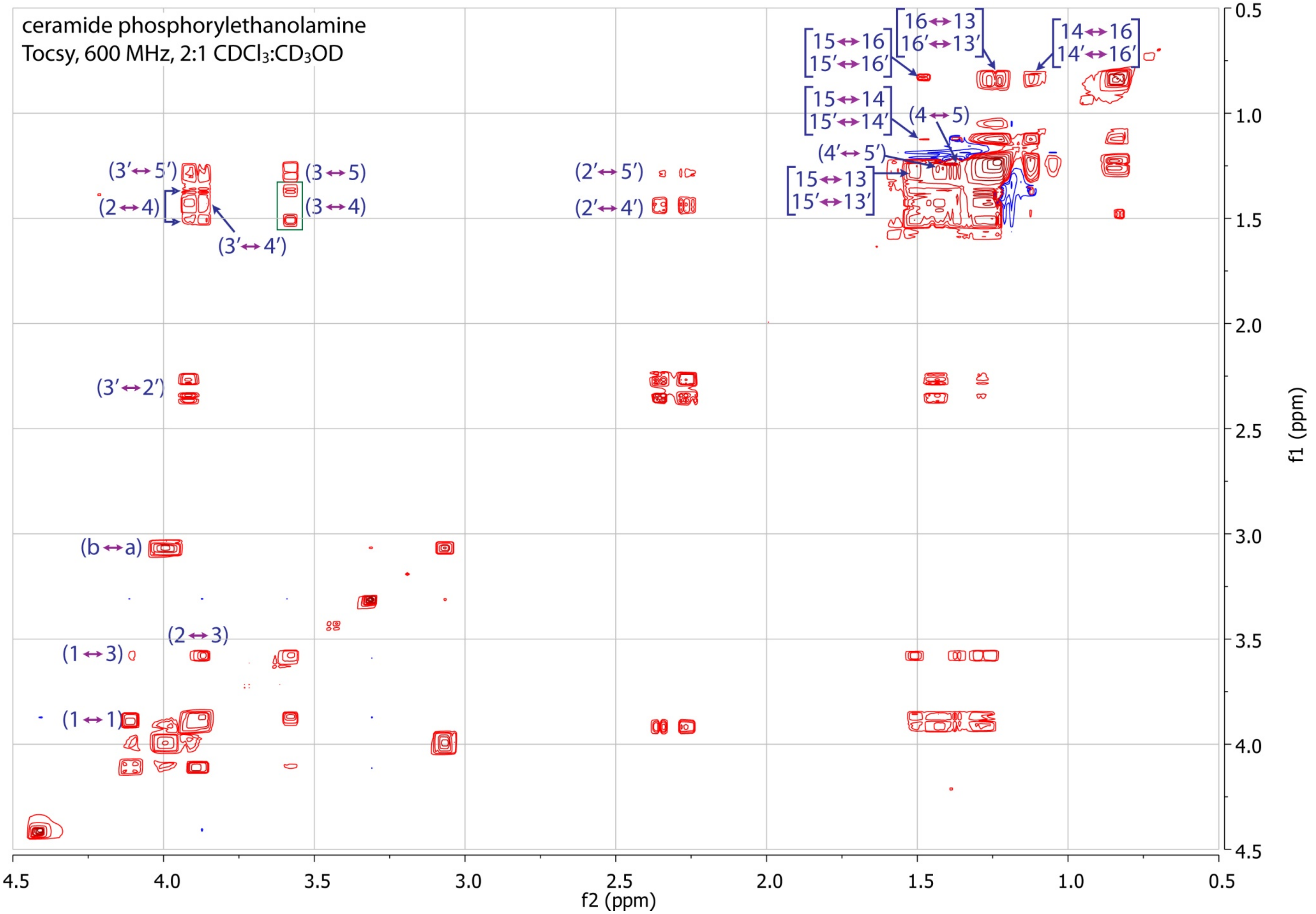
ceramide phosphorylethanolamine
Tocsy, 600 MHz, 2:1 CDCl3:CD3OD
(3′↔5′)
(2↔4)
(3′↔4′)
(3↔5)
(3↔4)
(2′↔5′)
(2′↔4′)
(3′↔2′)
(b↔a)
(2↔3)
(1↔3)
(1↔1)
15↔16
15′↔16′
16↔13
16′↔13′
14↔16
14′↔16′
15↔14
15′↔14′
(4↔5)
(4′↔5′)
15↔13
15′↔13′
f2 (ppm)
f1 (ppm)

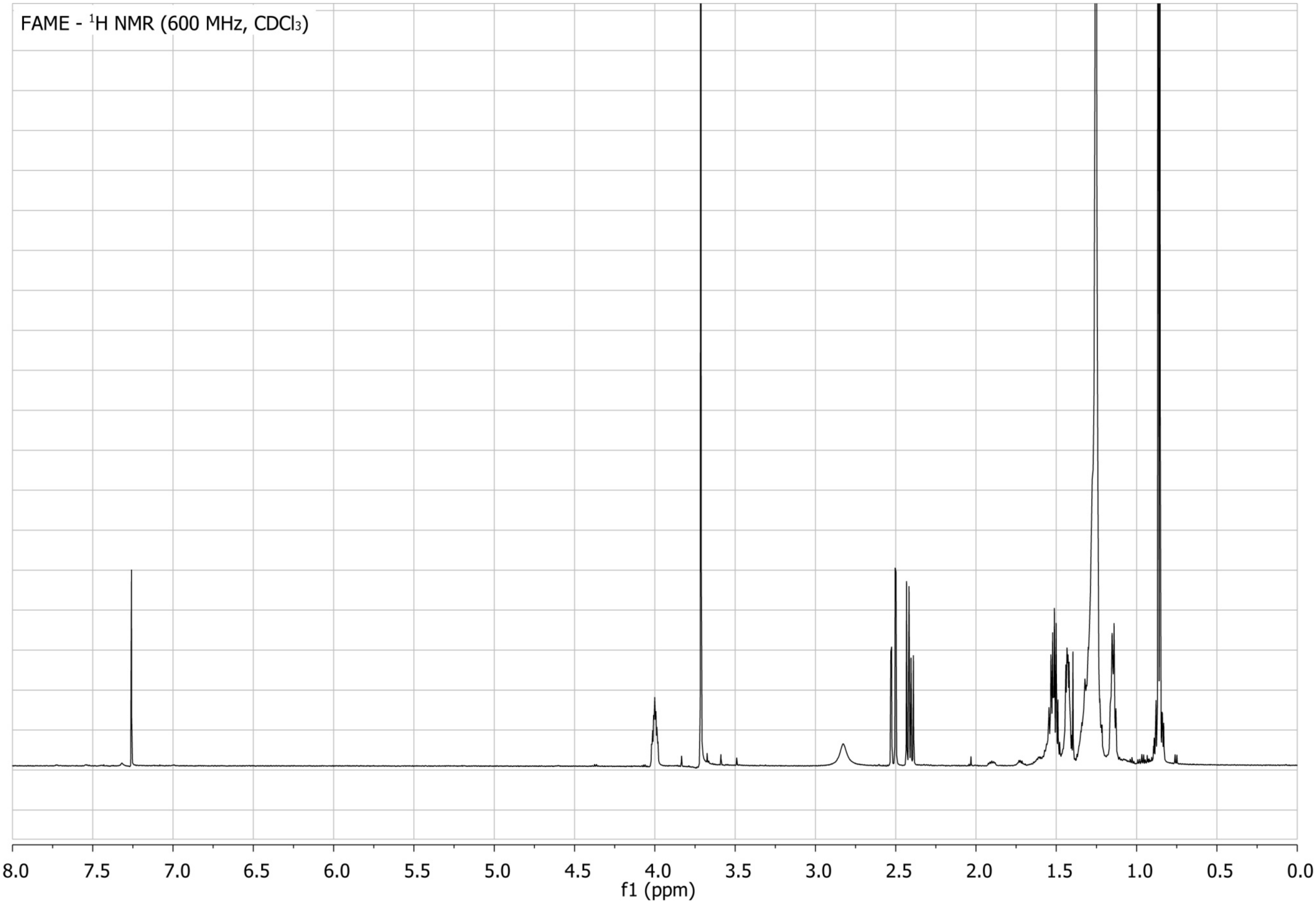
FAME - ^{1}H NMR (600 MHz, $CDCl_3$)
8.0 7.5 7.0 6.5 6.0 5.5 5.0 4.5 4.0 3.5 3.0 2.5 2.0 1.5 1.0 0.5 0.0
f1 (ppm)

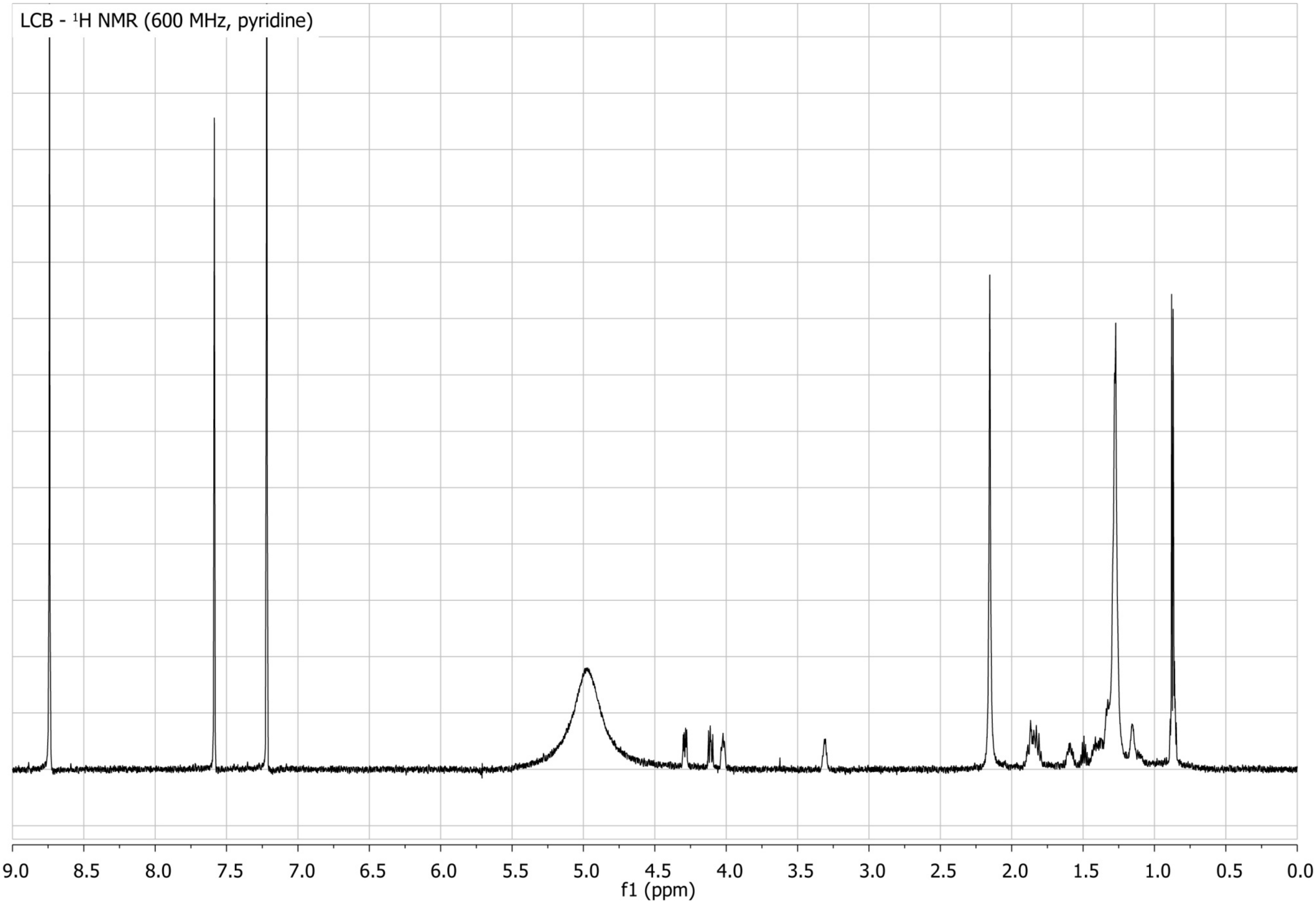

LCB - ¹H NMR (600 MHz, pyridine)
9.0 8.5 8.0 7.5 7.0 6.5 6.0 5.5 5.0 4.5 4.0 3.5 3.0 2.5 2.0 1.5 1.0 0.5 0.0
f1 (ppm)

S4.4. MS/MS data for α-GalCer$_{BF}$. MS/MS data was obtained on a 9.4 T Fourier Transform Ion Cyclotron Resonance (FTICR) Mass Spectrometer at the Yale Keck Biotechnology Resource.

MS/MS data for α-GalCer$_{BF}$

MS/MS data for [M-H] 716.57:

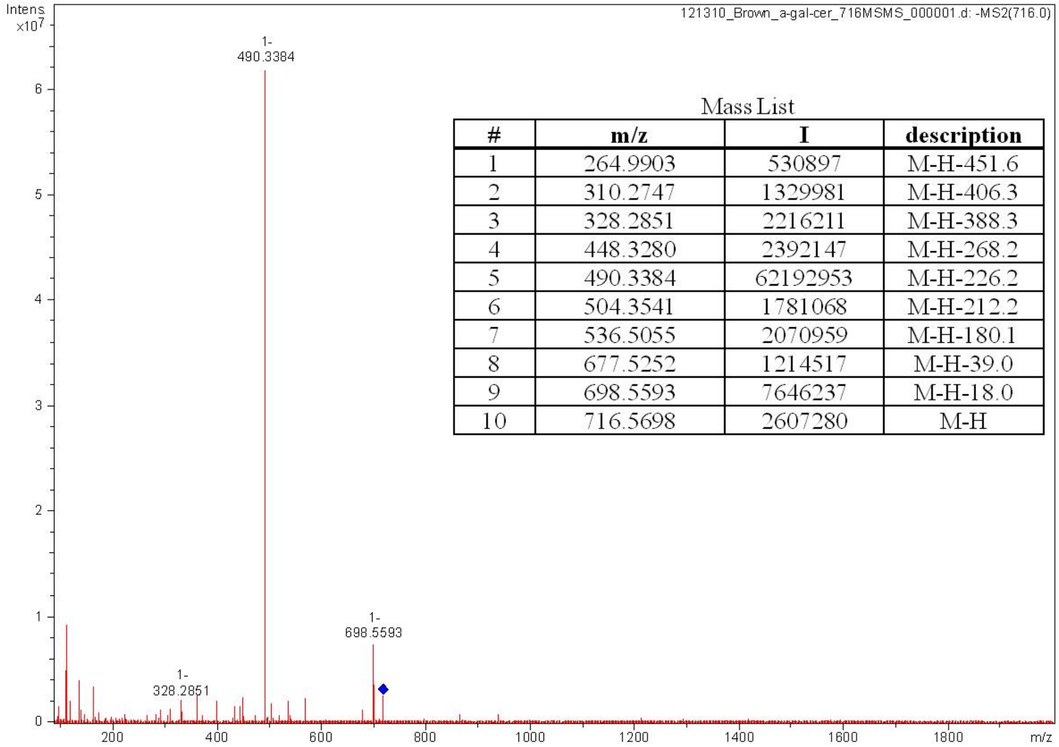

Mass List

#	m/z	I	description
1	264.9903	530897	M-H-451.6
2	310.2747	1329981	M-H-406.3
3	328.2851	2216211	M-H-388.3
4	448.3280	2392147	M-H-268.2
5	490.3384	62192953	M-H-226.2
6	504.3541	1781068	M-H-212.2
7	536.5055	2070959	M-H-180.1
8	677.5252	1214517	M-H-39.0
9	698.5593	7646237	M-H-18.0
10	716.5698	2607280	M-H

MS/MS data for [M-H] 730.59:

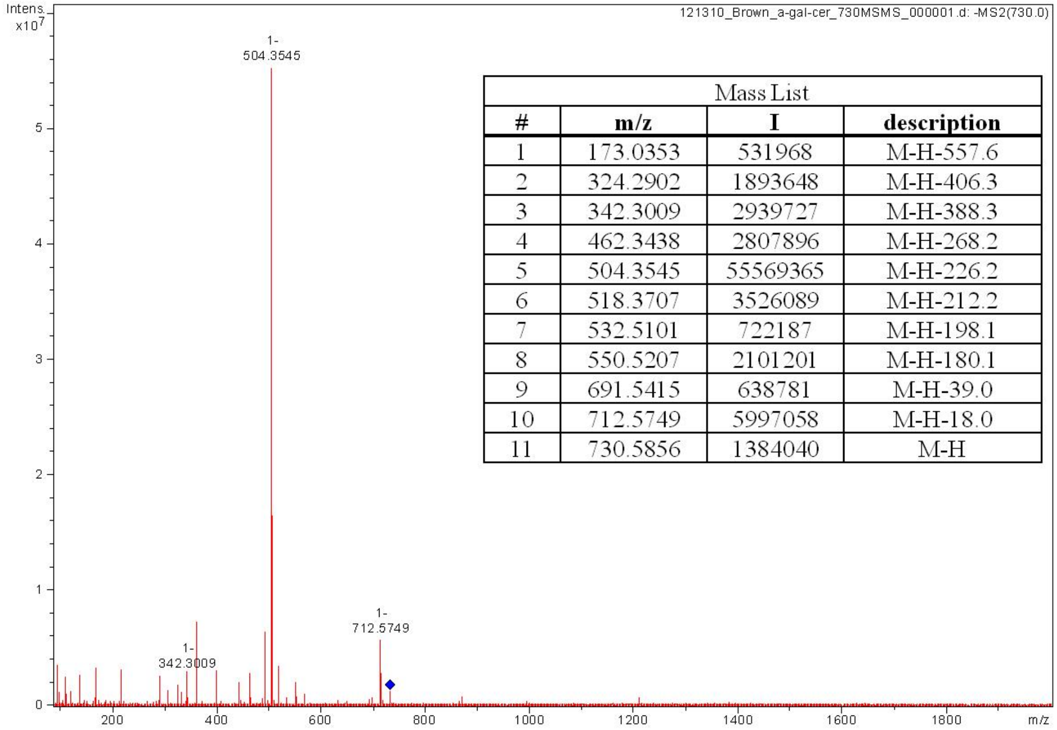

Mass List

#	m/z	I	description
1	173.0353	531968	M-H-557.6
2	324.2902	1893648	M-H-406.3
3	342.3009	2939727	M-H-388.3
4	462.3438	2807896	M-H-268.2
5	504.3545	55569365	M-H-226.2
6	518.3707	3526089	M-H-212.2
7	532.5101	722187	M-H-198.1
8	550.5207	2101201	M-H-180.1
9	691.5415	638781	M-H-39.0
10	712.5749	5997058	M-H-18.0
11	730.5856	1384040	M-H

Proposed fragment ion structures for [M-H] 716.57:[8]

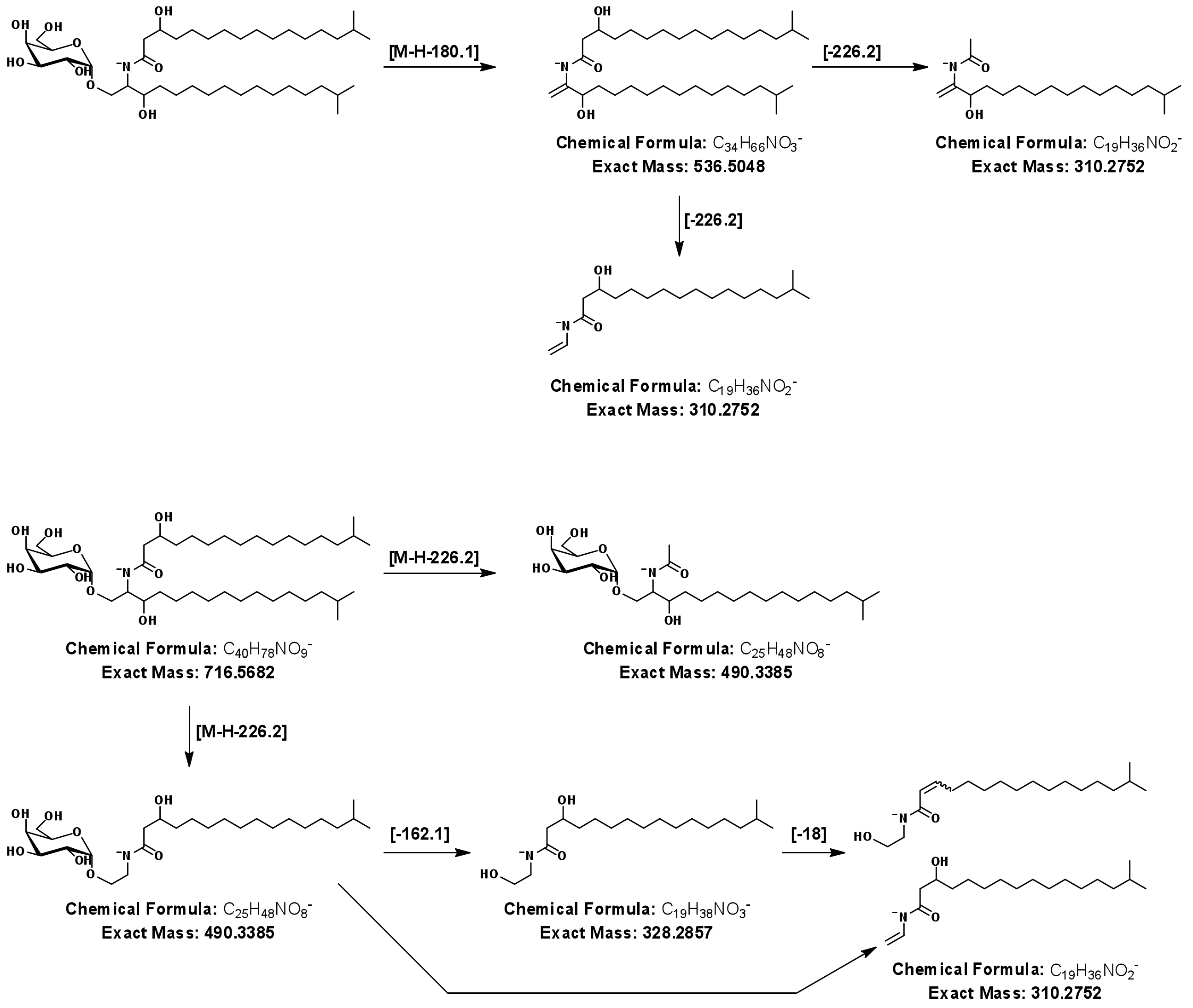

[8] Han, X. "Characterization and Direct Quantitation of Ceramide Molecular Species from Lipid Extracts of Biological Samples by Electrospray Ionization Tandem Mass Spectrometry" *Analytical Biochemistry* **2002**, *302*, 199–212.

S4.5. Overlaid 1H NMR data for CPE, ceramide$_{BF}$, and α-GalCer$_{BF}$. (600 MHz, 2:1 $CDCl_3$:CD_3OD) indicating >95% purity (see also the spectra on S15, S23, and S33). Magnified regions of the overlaid spectra from 3.40-4.20 ppm and 2.20-2.39 ppm, shown on the next two pages, indicate that α-GalCerBf is free from contamination by ceramide phosphorylethanolamine or ceramide.

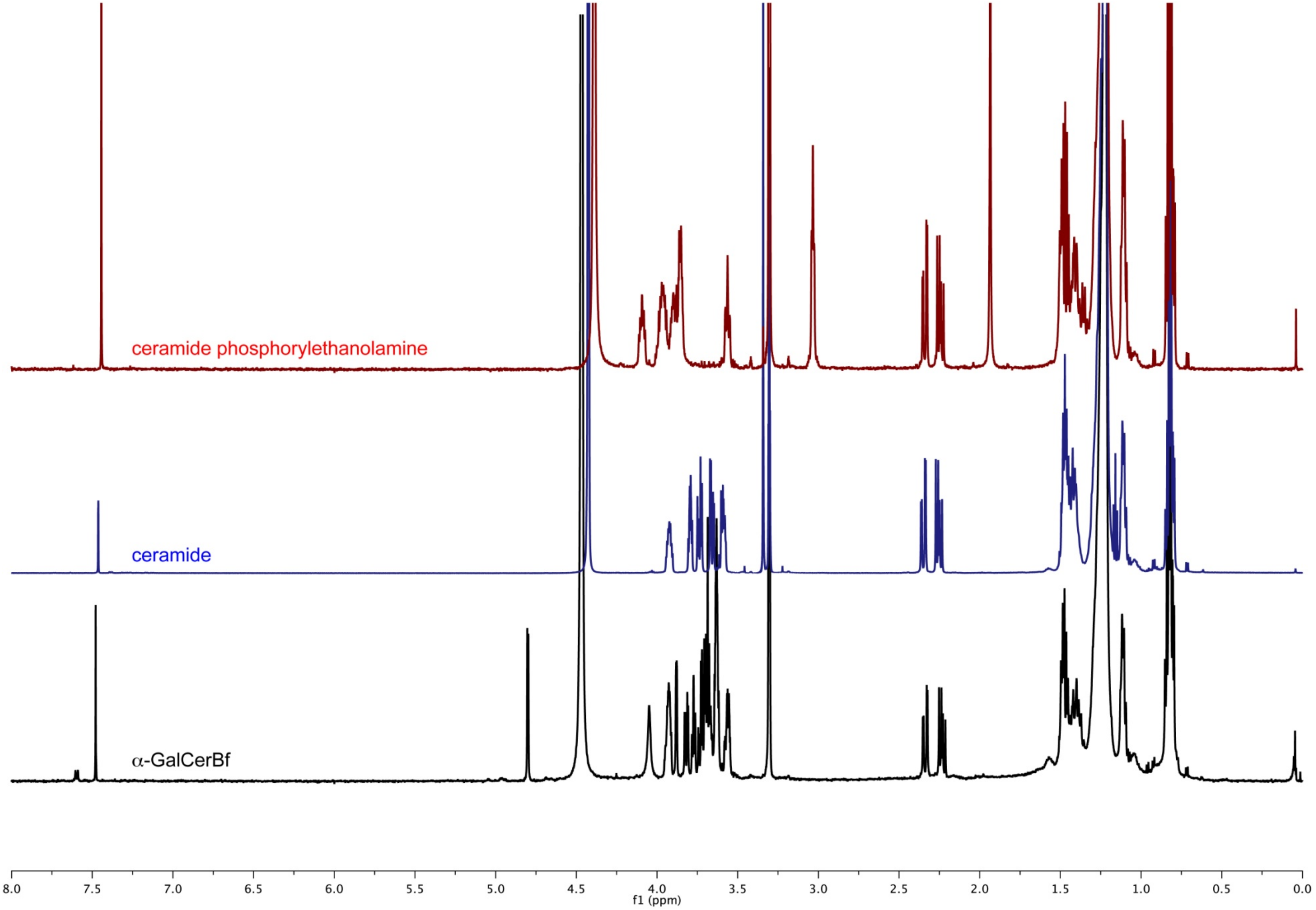

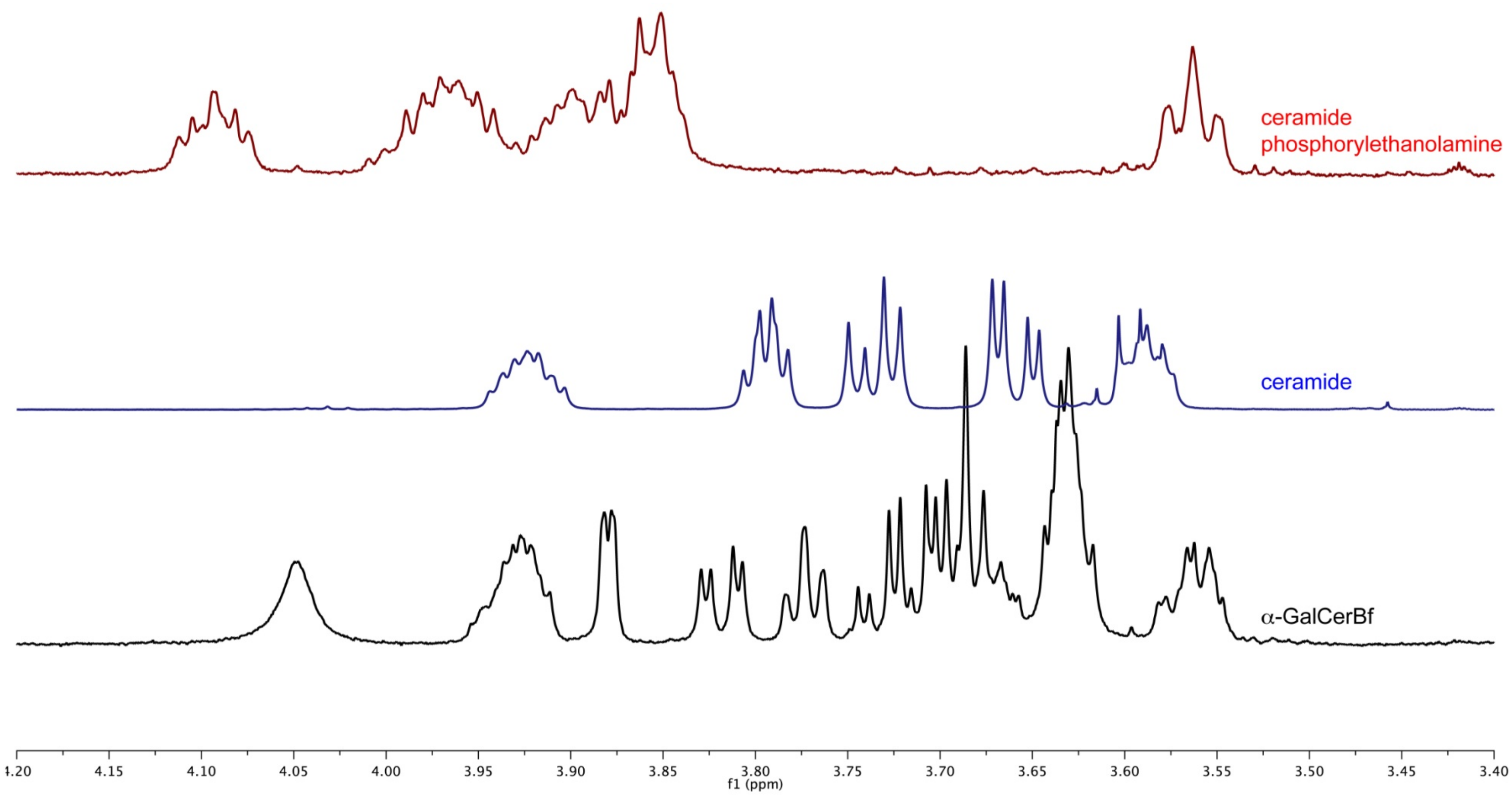
ceramide
phosphorylethanolamine
ceramide
α-GalCerBf
4.20 4.15 4.10 4.05 4.00 3.95 3.90 3.85 3.80 3.75 3.70 3.65 3.60 3.55 3.50 3.45 3.40
f1 (ppm)

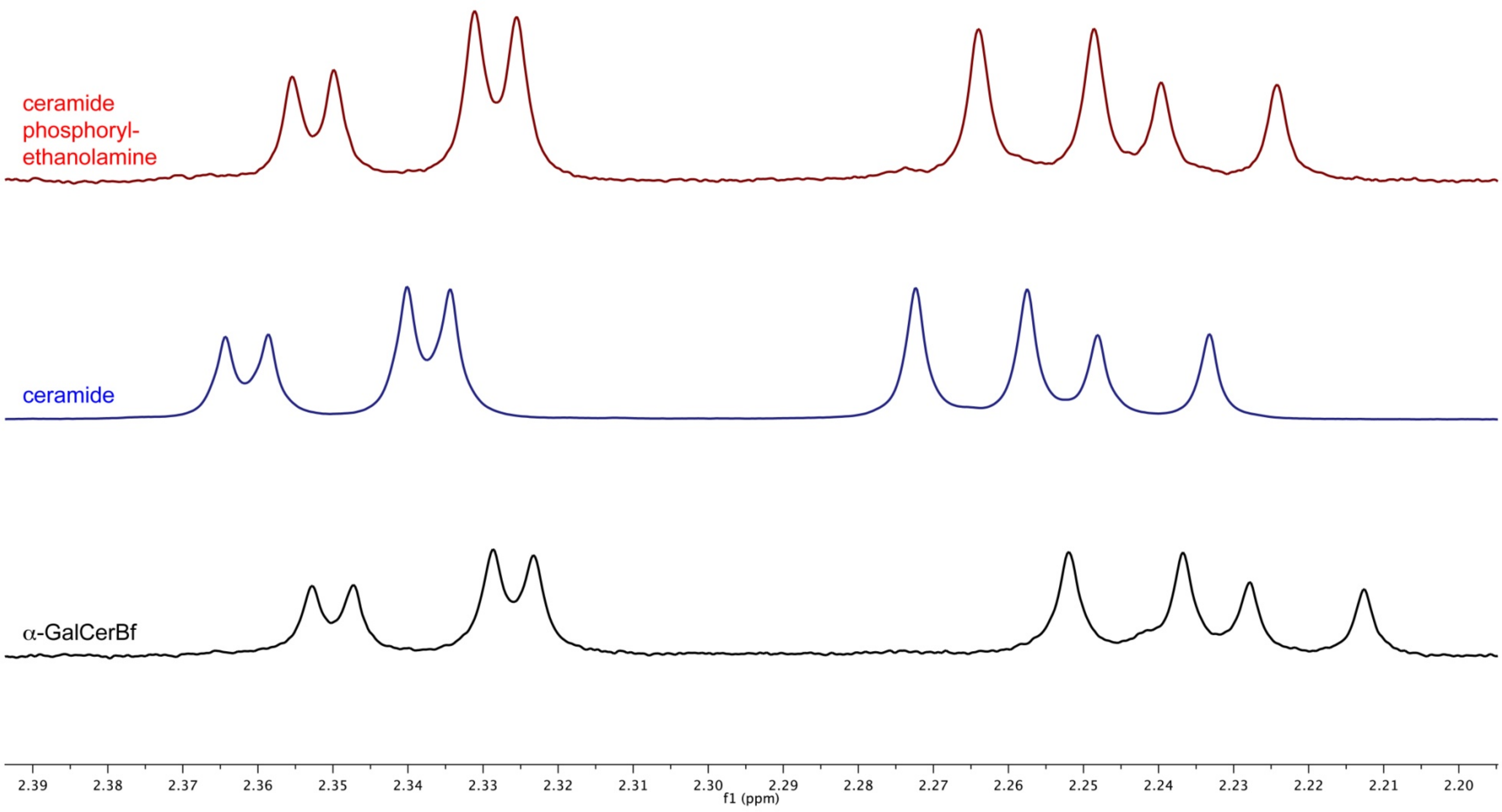
ceramide
phosphoryl-
ethanolamine
ceramide
α-GalCerBf
2.39 2.38 2.37 2.36 2.35 2.34 2.33 2.32 2.31 2.30 2.29 2.28 2.27 2.26 2.25 2.24 2.23 2.22 2.21 2.20
f1 (ppm)

***S4.6. Analysis of lipid tail length variation in ceramide$_{BF}$*.** Ceramide$_{Bf}$ was methanolyzed as described in *S1.11*, and the resulting long chain base (LCB) was analyzed by HPLC-MS. Three variants of the LCB were observed, differing in the lipid chain length as shown in the proposed structures below.

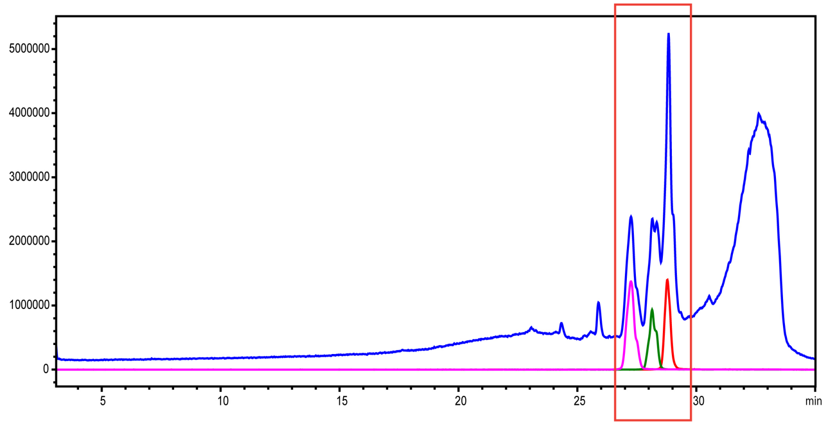

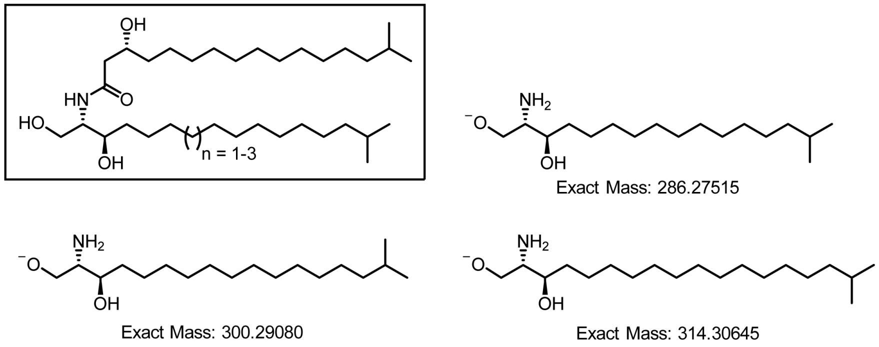

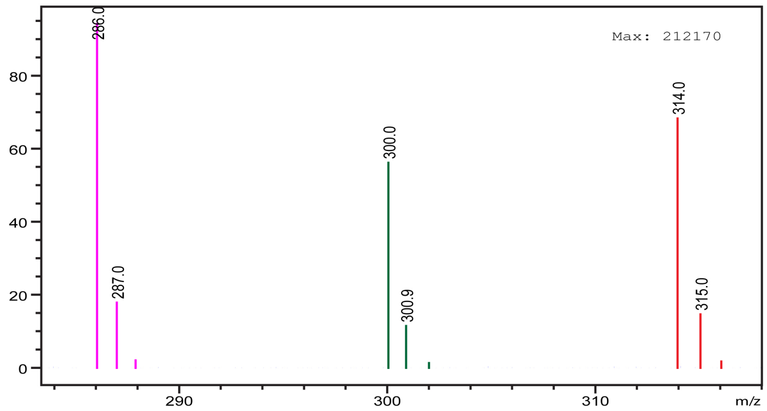

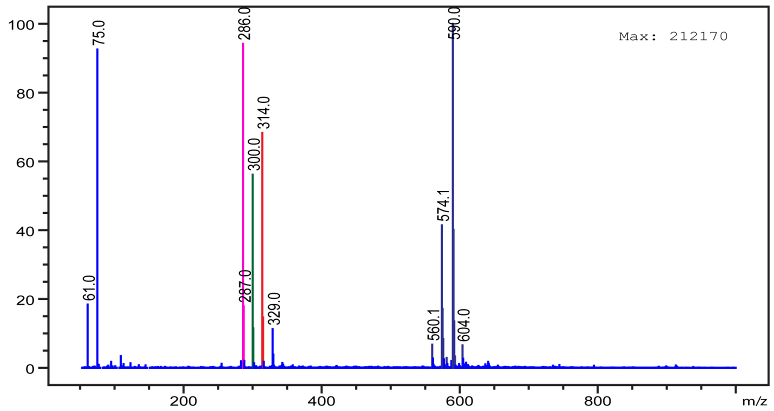

Supplement: Supporting Information S1 — Contents: Section S1, materials, equipment, and general methods. Section S2, high-resolution mass spectrometry and LC-MS analysis. Section S3, in vitro titration data. Section S4, spectral data. Figure S1, B. fragilis Δ2461 is deficient in the production of sphingolipids. LC-MS trace with extracted ions shown [M-H]. See Figure 2 legend for details. Figure S2, α-GalCerBf binds CD1d in vitro. Hybridomas were stained with anti-CD3 antibodies and empty mCD1d tetramers or mCD1d tetramers loaded with α-GalCerBf or KRN7000. Flow cytometry plots representative of three independent experiments are shown. (A) Plots show forward (FSC) and side (SSC) scatter of all events. (B) Plots pre-gated as shown in (A) and further gated on DAPI-negative events show staining with tetramer and CD3 antibodies. Figure S3, KRN7000 and α-GalCerBf titration in vitro. BMDCs and NKT hybridomas were cultured at a 3∶1 hybridoma:BMDC ratio and the indicated doses of KRN7000 or α-GalCerBf in the presence of 1 µg/ml LPS. Supernatants were harvested after 24 h and IL-2 production was measured by ELISA. Table S1, Primers used in this study. (PDF) [file pbio.1001610.s001.pdf]
